# Barriers and enablers shaping kidney transplant care delivery: Insights from Iraqi pharmacists

**DOI:** 10.1371/journal.pone.0360647

**Published:** 2026-10-12

**Authors:** Sarah M. Faisal, Samer Imad Mohammed

**Affiliations:** Department of Clinical Pharmacy, College of Pharmacy, University of Baghdad, Baghdad, Iraq; Universiti Monash Malaysia: Monash University Malaysia, MALAYSIA

## Abstract

**Background:**

Kidney transplant care in low-resource countries is often affected by multiple factors, including workforce constraints, fragmented communication, and medication access challenges. Given pharmacists’ involvement in medication management and regular patient follow-up, this study was designed to explore transplant care delivery in Iraq from pharmacists’ perspectives.

**Method:**

Qualitative data were collected using face-to-face, semi-structured interviews with all eligible pharmacists from the two major governmental hospitals in Baghdad caring for kidney transplant recipients. Afterwards, data were analyzed using Braun and Clarke’s reflexive thematic analysis approach.

**Results:**

This study included 13 pharmacists, 5 of whom were working in the clinical transplant wards, whereas the remaining 8 were working in the outpatient consultation setting. Four descriptive themes were identified: Dispensing-centered practice within a constrained and evolving pharmacist role, fragmented yet adaptive interprofessional collaboration, patient education as an informal and inconsistently delivered practice, and medication availability within a constrained and variably regulated supply system.

**Conclusion:**

This study highlights the adaptive approaches used by pharmacists in an attempt to maintain medication access and patient support with the available resources. However, the study findings also highlight challenges that extend beyond the capacity of individual pharmacists to resolve and suggest the need for more structured organizational support, clearer communication pathways, and an integrated system to strengthen Iraqi transplant care practices within comparable governmental settings.

## 1. Introduction

Kidney failure, or end-stage renal disease (ESRD), is a serious medical issue that requires prompt treatment. Dialysis and renal transplantation are the main modalities used to address this condition. While dialysis can help with the symptoms of ESRD, transplantation is the preferred treatment in most cases, as it improves survival and quality of life compared to long-term dialysis, as shown by a large systematic review of 110 studies comparing transplantation to different dialysis modalities across different health care systems [1].

However, the success of transplantation extends far beyond the surgery itself, as transplantation is a life-changing event that requires continuous follow-ups and a healthy lifestyle to protect the new kidney from organ rejection and infections and to help the patient adapt to their lifelong immunosuppressant regimen [2]. For that purpose, guidelines such as Kidney Disease: Improving Global Outcomes (KDIGO) have emerged to organize the management of kidney transplant recipients in the early postoperative phase and the later phase [3].

Globally, kidney transplantation is usually managed by a multidisciplinary team that involves a transplant pharmacist whose role has evolved from traditional dispensing to involve multiple clinical roles before and after transplantation. These responsibilities include identifying drug-drug and drug-disease interactions; managing immunosuppressant doses and possible toxicities; and providing patient education pre- and post-transplantation to improve adherence and minimize unplanned emergency department visits [4].

Although previous studies have focused on patient-related factors affecting transplant outcomes, such as patient income, education, and transportation [5], limited studies have been conducted to explore how care is delivered in real-world practice, especially in low- and middle-income countries (LMICs). In such settings, kidney transplantation may be affected by broader system-related challenges, including infrastructure limitations, financial constraints, workforce shortages, and disparities in access to transplant services. Collectively, these factors may influence how transplant services are organized and delivered in routine clinical practice [6].

Despite increasing interest in transplant care in LMICs, limited data existed regarding transplant healthcare delivery in Iraqi settings. Pharmacists can provide a distinct perspective on care delivery because of their central role in medication management and their interactions with both patients and the healthcare providers serving them, as well as their involvement in routine visits as a point of medication supply. As points of medication dispensing, pharmacists maintain regular contact with kidney transplant recipients, including those who usually receive routine clinical follow-up in private clinics but continue to obtain immunosuppressive medications from governmental hospitals. All of these factors enable pharmacists to better identify medication- and system-related challenges affecting care delivery.

Therefore, the study aimed to explore kidney transplant care in governmental hospitals in Iraq from pharmacists’ perspectives by describing current practices, identifying key barriers and enablers, and capturing pharmacists’ suggestions for improvement opportunities.

## 2. Materials and methods

### 2.1. Study design and setting

This study used a qualitative descriptive approach, chosen to provide a direct description of pharmacists’ perspectives on health care delivery to kidney transplant recipients and their experiences within these settings. Semi-structured interviews using open-ended questions were conducted to investigate current practices, barriers and enablers influencing care delivery, along with participants’ recommendations for potential system enhancements. In addition to the interview questions, question prompts were used to achieve a thorough understanding of participants’ perspectives.

The concept of the interview guide was initially formed by preliminary informal discussions with pharmacists to gain a better understanding of common difficulties and standard practices. Pharmacists’ responses were then used to shape the main study objectives, which were subsequently used to develop the interview guide. The guide was then reviewed and refined by seven academic experts from the clinical pharmacy faculty members, all of whom possess substantial experience with qualitative studies, as well as a board-certified clinical pharmacist and a urologist specialized in kidney transplantation. All of their comments were reviewed by the two authors, and suitable adjustments were made to generate the final version of the guide.

The guide focused on seven main domains: the scope of pharmacists’ responsibilities toward kidney transplant recipients, the pharmacist’s educational role toward transplant patients, collaboration and data sharing among healthcare providers, medication availability and access, barriers to optimal care delivery, suggestions for improvement, and a specific inquiry directed at outpatient pharmacists regarding the unique challenges faced by external patients. These domains were explored using twelve questions designed to help answer the study questions and provide a clearer understanding of real-world practices. The Arabic and English versions of the interview guide are provided in S1 and S2 Files, respectively.A formal pilot of the interview guide was not conducted because the study population was limited to pharmacists working in the two major governmental hospitals providing kidney transplant services in Baghdad. Given the small number of eligible participants, all eligible pharmacists were included in the main study to maximize representation of the target population.

This study was conducted between 25/11/2025 and 20/2/2026 in the two major governmental hospitals in Baghdad that care for kidney transplant recipients. The governmental hospitals were selected because they serve as the primary locations for pharmacists involved in the care of transplant recipients, which facilitated their recruitment. Moreover, governmental hospitals accommodate pharmacists working in different settings, caring for both inpatients and outpatients, which would allow a more comprehensive exploration of their roles and experiences in transplant-related care. Furthermore, governmental hospitals are responsible for transplant medication provision, which would allow investigation of pharmacists’ contributions to patients’ routine follow-ups.

Both of the selected hospitals had an inpatient transplant ward, where kidney transplantation was usually performed, each of them staffed with physicians, nurses and pharmacists serving those patients.

However, the organization of the transplant outpatient services differed between the two hospitals. In one hospital, there was no dedicated office or center for outpatient transplant services; instead, these services were delivered within general outpatient services, and the physician providing this service usually had other clinical responsibilities elsewhere, while transplant medication was typically provided through a designated line within a shared outpatient pharmacy.

At the other hospital, outpatient follow-up was delivered through a dedicated transplant center, where physicians were assigned to serve those patients during clinic hours. Transplant medications were provided through an outpatient pharmacy within the center that was dedicated to those patients, so all relevant services were located in close proximity.

### 2.2. Participants and recruitment

This study included pharmacists from both inpatient and outpatient settings given their direct involvement in transplant care delivery. Inpatient pharmacists are typically involved in immediate postoperative care, while outpatient pharmacists usually play a central role in routine follow-up visits through medication management. This variation in settings allowed representation of different stages of transplant care delivery.

Therefore, eligible participants were pharmacists working in the inpatient transplant ward and those working in the outpatient pharmacy who had direct contact with transplant patients as part of their routine work and were available during the study period. A total population sampling approach was used, whereby all eligible pharmacists working in the selected setting were invited to participate. Sixteen pharmacists met the eligibility criteria, but only thirteen pharmacists participated in this study, as one pharmacist declined participation and two others were unavailable due to extended leave.

Recruitment therefore ended after interviewing all available eligible pharmacists, announcing the end of data collection.

### 2.3. Data collection and analysis

Data were collected through face-to-face semi-structured interviews by the first author, who is a pharmacist and a master’s candidate in the clinical pharmacy department with approximately five years of experience. The first author had no prior professional or supervisory relationship with the study participants before conducting the interviews. However, the interviewer’s professional background as a pharmacist facilitated communication and rapport with participants. The use of open-ended questions and a response-clarification approach, along with discussing code and theme development with the second author, was intended to minimize potential interviewer and analytical bias.

All interviews were carried out in Arabic and took place in a quiet private room within the participating hospitals to ensure privacy and minimize interruptions during the interviews. Interviews were audio-recorded after obtaining participants’ consent. During the interviews, the first author periodically summarized and paraphrased participants’ responses to confirm understanding and obtain clarification as needed; each interview lasted approximately 20–40 minutes. Afterwards, the recordings were manually transcribed verbatim and de-identified by the first author, then de-identified transcript excerpts were translated into English with the assistance of ChatGPT (OpenAI). The first author subsequently reviewed each translated section against the original Arabic transcript to ensure conceptual accuracy and meaning preservation. The second author then revised both the Arabic and English transcripts before coding.

To address the study objective, the collected data were analyzed using Braun and Clarke’s reflexive thematic analysis [7], as this approach is well suited to capturing themes and patterns across participants’ responses. The analysis was primarily inductive, as codes were generated from the collected data rather than from a predefined coding framework; it also focused on the explicit meaning described by participants rather than investigating latent meanings. Notably, the broad areas of analysis were initially guided by the interview guide, such as pharmacists’ role, education, collaboration, etc.; however, the names of the themes and the subthemes, as well as their content, emerged explicitly from the collected data.

The analysis proceeded through several iterative steps. First, the transcripts were reviewed repeatedly to develop familiarity with the data. Then, the first author performed initial coding using the English-translated transcripts. Afterwards, these codes were reviewed, merged and renamed as needed; then similar codes were grouped to develop the preliminary subthemes and themes.

The initial Codes and developing themes were reviewed and discussed with the second author at different stages of the analysis process to ensure clarity and reduce the influence of individual interpretation. Differences in interpretation were resolved through discussion; themes and subthemes were iteratively reviewed and refined to ensure accurate representation of the data until the final thematic structure was agreed upon.

Throughout the study, QDA Miner Lite software supported data management and organization.

### 2.4. Ethical considerations

Validation for this study was obtained from the Scientific and Ethical Committee in the College of Pharmacy, University of Baghdad (approval number RECAUBCP289202506H). Additional approval was obtained from the Ministry of Health (MOH), including permission from the relevant hospital authorities to conduct the study within the selected settings.

Prior to each interview, participants were explicitly informed about the purpose of the study, and informed verbal consent was obtained. The use of verbal consent was considered appropriate given the minimal-risk nature of the study, which consisted of face-to-face interviews with healthcare professionals and did not require the collection of any sensitive personal information or any kind of intervention. This approach was also used to facilitate participation by avoiding unnecessary administrative procedures, and this practice was approved by the Scientific and Ethical Committee in the College of Pharmacy, University of Baghdad, as part of the study protocol.

To document Consent, participants confirmed both their participation and audio recording at the beginning of each interview, and these confirmations were retained within the interview recordings. Participants were also assured that the interviews would be anonymized and that all information would be treated confidentially and used for research purposes only.

## 3. Results

### 3.1. Participant characteristics

Thirteen pharmacists participated in this study, five of whom were males and eight were females. In terms of the study setting, five pharmacists were working in the clinical transplant wards, whereas the remaining eight pharmacists were working in the outpatient consultation setting. The median experience of those pharmacists was two years, with a range from one month to six years of practice in a transplant setting.

Participants were recruited from the two major governmental hospitals in Baghdad. For confidentiality purposes, the hospital names were anonymized. Detailed participant characteristics are presented in Table 1.

**Table 1:** Characteristics of individual pharmacist participants.

Participant ID	Sex	Practice Setting	Hospital	Years of Experience in Transplant Setting
Ph1	Female	Inpatient	Hospital 1	1 Month
Ph2	Male	Outpatient	Hospital 1	3 Years
Ph3	Female	Outpatient	Hospital 1	2 Years
Ph4	Female	Outpatient	Hospital 1	3 Years
Ph5	Female	Inpatient	Hospital 1	1 Month
Ph6	Female	Outpatient	Hospital 1	7 Months
Ph7	Male	Outpatient	Hospital 1	5 Years
Ph8	Female	Outpatient	Hospital 2	4 Years
Ph9	Male	Outpatient	Hospital 2	6 Years
Ph10	Female	Inpatient	Hospital 2	4 Months
Ph11	Female	Inpatient	Hospital 2	3 Years
Ph12	Male	Inpatient	Hospital 2	1.5 Years
Ph13	Male	Outpatient	Hospital 1	4 Months

### 3.2. Main descriptive themes

Following the analysis of the qualitative data, four descriptive themes were identified; these themes described the current practices of pharmacists in the kidney transplant care setting, emphasizing both strengths and areas of challenge.

Although the emerging themes reflected the areas initially intended to be explored in the interview guide, the analysis identified several additional findings that were not predefined in these domains, such as pharmacists’ involvement in activities beyond their assigned responsibilities, adaptive informal communication strategies, and the effect of organizational differences on care delivery.

#### 3.2.1. Dispensing-centered practice within a constrained and evolving pharmacist role.

This theme reflects the dominant nature of the pharmacist’s role in transplant practice; most participants (n = 9) described their responsibilities as primarily centered around dispensing, which appears to be evident in both inpatient and outpatient settings.

In addition to dispensing, administrative responsibilities appear to form a core component of pharmacists’ daily routine, especially for the outpatient pharmacists (n = 6). These responsibilities encompass organizing the issuance of a chronic disease booklet that enables patients to receive their medication on a monthly basis, as well as electronically archiving the patient file and documenting each medication dispensing process for that patient.

Another important task that was highlighted by a specialized unit linked to the outpatient pharmacy is dispensing planning and medication management. Ph7 mentioned, *“Monthly and yearly statistics are prepared by the pharmacists of the specialized chronic disease unit, as they hold all patient files”.*

Beyond dispensing and administrative work, several Participants described engaging in practices outside their formally assigned duties. For example, some pharmacists reported routinely reviewing prescriptions with patients and identifying possible prescription errors (n = 6). *“Although reviewing prescriptions with the patient is not officially part of our responsibilities, this practice has proved effective”* (ph4). Further evolving roles were described by more experienced pharmacists, such as improving medication stability through practical approaches gained through experience, identifying patterns in patients’ complaints, and reporting repeated and concerning medication adverse effects to the pharmacovigilance center. In addition, one experienced pharmacist responsible for administrative duties reported using monthly and annual patient and dispensing statistics to refine medication need estimation, which they perceived as contributing to improved medication supply.

Despite these contributions, all inpatient pharmacists agreed on the limitations of the pharmacist’s clinical role and restricted participation in decision-making within the patient management process. Ph1 stated, *“Reviewing laboratory data or making clinical decisions based on investigations is considered outside the scope of pharmacists’ authority in this department.”*

Another key issue participants emphasized was the lack of structured training and the reliance on senior colleagues and experience-driven knowledge for learning. Ph11 noted, *“Although we studied these medications at university, we did not study them with this level of detail.”*

Ph4 stated: *“Currently, newly appointed staff members learn from senior colleagues. However, this situation raises an important question: whether the knowledge and practices passed on by senior staff are always accurate and up-to-date.”*

Participants also linked the absence of structured training and frequent pharmacist rotation to variability in clinical and practical experience among pharmacists and to difficulties in developing experienced pharmacists within transplant care. Ph5 stated: *“Continuous staff rotation often results in newly assigned pharmacists, who possess general knowledge but lack the practical experience.”*

#### 3.2.2. Fragmented yet adaptive interprofessional collaboration.

The collaboration between healthcare providers appeared to differ depending on the practice setting and the organizational structure within each hospital. Participants described different patterns of collaboration between inpatient and outpatient settings, with several factors influencing communication and workflow.

In the inpatient setting, all pharmacists described frequent collaboration with nursing staff, which they considered particularly important for maintaining daily workflow and medication-related processes. Ph1 mentioned, *“Collaboration is primarily with the nursing staff, and this collaboration is strong and effective.”*

In contrast, all inpatient pharmacists described the pharmacist–physician communication as indirect and limited, perceiving it as a barrier affecting workflow and coordination. Ph11 stated, *“There is no direct communication between physicians and pharmacists. In my opinion, this limits opportunities for effective communication and could otherwise lead to improvements in the quality of services”*. Participants explained that communication with physicians usually occurred through nursing staff, who were described as a link between the two professions.

In contrast, Outpatient pharmacists described relatively more direct communication with physicians, although this communication was still considered limited because of workload pressure. Despite this, participants generally felt that the existing communication was sufficient to maintain workflow when needed. Ph3 noted, *“As for physicians, communication is present but relatively limited due to their workload. However, essential coordination does occur when needed, particularly in matters affecting continuity of care and medication management.”*

Participants have also described noticeable differences in communication patterns between the two hospitals. Participants at the hospital with a dedicated transplant center described smoother communication and easier coordination. Ph8 stated, “Communication usually takes place verbally and personally, as the physicians’ offices are located very close to the outpatient pharmacy.”

In contrast, participants from the hospital without a dedicated outpatient transplant center described greater communication difficulties, where healthcare providers were distributed across different areas within the hospital. Ph13 mentioned, *“the communication is somewhat limited, as physicians do not have a permanent room within the outpatient clinic where we can easily find them when needed.”*

Participants from this hospital described using several adaptive and informal communication methods. Outpatient pharmacists particularly highlighted the use of WhatsApp groups and telephone communication to coordinate medication availability and dispensing procedures. Similarly, one inpatient pharmacist reported the use of telephone communication to address urgent issues when direct communication was unavailable. However, only one pharmacist expressed concern about overreliance on these informal communication strategies, noting that they may not always provide reliable access to healthcare professionals when needed.

Several participants highlighted factors that facilitated collaboration, including experienced nursing staff, workflow coordination, monthly meetings, and mutual respect between healthcare providers. Ph10 stated*, “The presence of a specialized and experienced nursing staff facilitates cooperation and information exchange and helps the workflow proceed more smoothly.”* Outpatient pharmacists also described monthly meetings as important for coordinating dispensing strategies and reviewing medication stock levels. Ph13 mentioned, *“At the beginning of each month, medication supply and dispensing strategies are discussed.”*

Interpersonal relationships were also perceived to influence collaboration. Mutual trust, respectful communication, and quick physician responses were described as factors that supported pharmacists when dealing with patient-related issues (n = 6). However, a few pharmacists (n = 3) believed that age differences and professional hierarchy could occasionally contribute to resistance toward pharmacists’ recommendations. Ph12 stated*, “I believe that age differences may represent one challenge, as some older nurses may not easily accept information from younger pharmacists.”*

#### 3.2.3. Patient education as an informal and inconsistently delivered practice.

Most pharmacists reported being involved in the education process. However, this involvement varied among pharmacists and across various practice settings. In most cases, patient education focused mainly on medication. In contrast, few pharmacists reported educating patients on broader topics such as self-care, infection prevention, and nutrition, in addition to emphasizing the importance of regular follow-up appointments and medication adherence.

The approach to educational delivery also varied among participants. Most participants (n = 8) reported giving verbal education, while some pharmacists (n = 4) reported providing written instructions to patients. On the other hand, only two pharmacists mentioned indirect patient education through the nursing staff; this indirect educational approach was provided either by writing instructions on the medication package or informing the nursing staff, and then the latter takes on patient education responsibilities. Ph1: *“The nursing staff is responsible for patient education, as they are familiar with most of the required details.”*

Discharge counseling was also reported among inpatient pharmacists; 3 of 5 pharmacists reported reviewing discharge prescription sheets, reinforcing patient information, and providing adherence-aid strategies.

In the outpatient setting, several outpatient pharmacists also described their educational role as reinforcing the physician’s instructions, as patients usually return to the pharmacy seeking additional reassurance and clarification regarding their prescribed medication*.*

Ph3: *“Although most of the essential information is initially explained by the physician, many patients seek confirmation and reassurance.”*

In terms of assessing patient understanding, formal assessment was reported to occur less frequently, with only two pharmacists reporting the use of the teach-back method (asking patients to repeat the provided instruction). One pharmacist linked the limited use of these practices to time constraints and workload pressure. Ph7: *“Due to the heavy workload at the dispensing window. We cannot* *spend a long time with each patient. Therefore, we usually limit ourselves to providing the essential instructions briefly and directly.”*

In addition to this method, a few pharmacists (n = 3) reported using other strategies that may facilitate patient understanding, like involving caregivers in the education process and reinforcing information through shared listening at the dispensing window.

Despite these efforts, pharmacists reported differences in patients’ receptiveness toward education. Some patients were described as cooperative and interested in receiving medication-related education. However, a few other patients showed resistance and a lack of interest in the pharmacists’ counseling or follow-up instructions.

#### 3.2.4. Medication availability within a constrained and variably regulated supply system.

Most pharmacists reported consistent medication availability for transplant patients. However, temporary shortages were also reported, while prolonged complete shortages were described as uncommon.

The nature of these shortages was described as reduced dispensing quantities or unavailability of specific medication strengths. Participants identified several factors contributing to these shortages, including inconsistent medication supply, using outdated need estimation reports, and increasing medication demand as a typical consequence of increasing the number of transplant patients.

Pharmacists described multiple strategies that are commonly used to manage medication shortages. The most widely reported strategy was adjusting dispensed quantities to ensure fair distribution among patients. PH2: *“In cases of medication shortages, we are informed accordingly, and the allocated quantity per patient is adjusted to ensure equitable distribution.”*

Other strategies were also reported, such as reallocating medications between hospitals and maintaining an emergency stock in the pharmacy of the transplant ward. Ph11*: “We usually keep a reserved stock known as an ‘emergency supply’ in anticipation of any unexpected shortages.”*

The final option would be to refer the patient to a physician to explore the possibility of switching to an alternative medication, or ultimately, to advise the patient to purchase the medication from local pharmacies.

Beyond shortages, participants also described challenges related to fragmented follow-up care, weak communication with external physicians, and the absence of a unified system linking dispensing points. Two pharmacists expressed their concerns about possible duplicate dispensing practices due to this system gap, as patients could issue two chronic disease booklets at two dispensing points.

Ph12 stated: *“The absence of a unified system linking the different dispensing points may allow medication leakage, as a patient could open multiple booklets at different dispensing points and receive more than one medication supply.”*

Outpatient pharmacists also reported encountering outdated reports or unclear prescriptions, particularly among patients who underwent their surgery in other provinces or countries. Participants perceived long distances and financial issues as possible causes of these situations because it is difficult for those patients to obtain the required clarification or updated report. Participants described this as increasing pharmacists’ workload and causing frustration and dispensing delays for patients.

Beyond system and dispensing-related challenges, several pharmacists mentioned challenges associated with frequent changes in medication manufacturers; these changes appeared to create both patient-related and staff-related challenges. Six pharmacists described patients’ concerns and confusion when medication manufacturers were changed; one pharmacist reported that some patients may skip doses until they are reassured by their private physician about the new manufacturer. Ph11 stated*: “Patients often become anxious about using medications from a different company, and in some cases, they may even skip doses until they verify the new product and feel reassured about it.”*

Two Participants also highlighted that this variability could create practical challenges in medication preparation and administration, while one pharmacist perceived it as a source of medication error due to confusion about drug concentration.

ph12: *“Anti-thymocyte globulin (ATG) is available from two different companies. These are supplied in vials with different concentrations. Some staff assumed that both products were identical and could be administered in the same way. However, this confusion has, in some cases, led to dosing errors.”*

### 3.3. Summary of barriers and enablers influencing transplant care practices

Participants described multiple factors affecting pharmacy practices for transplant patients. These factors were synthesized from the thematic findings and participants’ responses to the interview questions specifically designed to explore barriers and enablers within the current transplant care system. A conceptual summary of these factors is presented in Fig 1.

**Fig 1:**
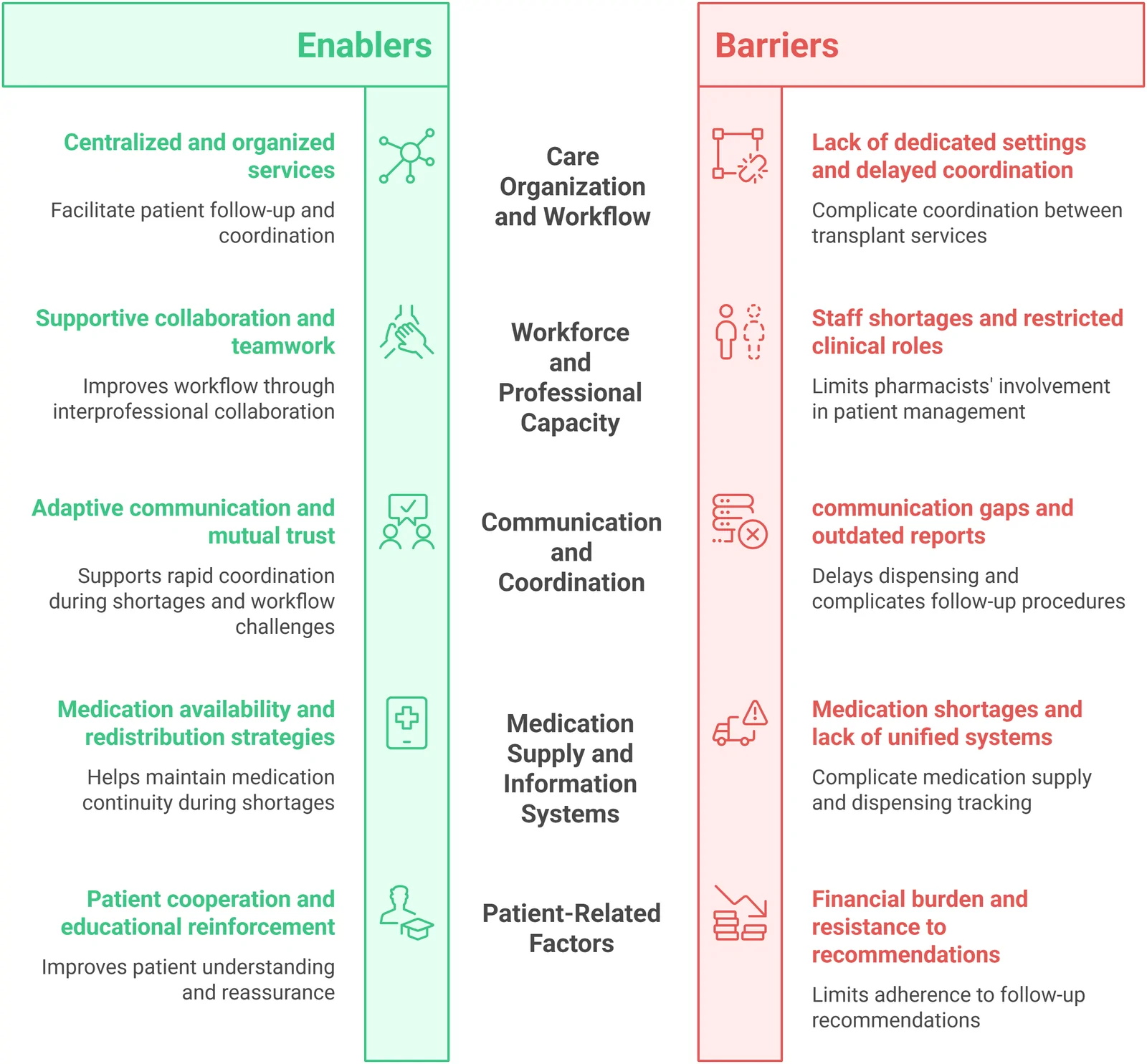
Conceptual overview of the main barriers and enablers mentioned by pharmacists in transplant units.

### 3.4. Participants’ suggestions for improvement

At the end of each interview, participants were asked about suggestions to improve the transplant pharmacy practice and continuity of care. The participants’ main suggestions for improvement are summarized in Table 2.

**Table 2:** Pharmacists’ recommendations to improve transplant care.

Practice challenge	Suggested improvement
Restricted pharmacist role, administrative burden, and limited specialized training	Expand pharmacists’ clinical role through active integration in ward rounds and clinical decision-making, reduce non-clinical administrative duties, and organize structured training programs regarding immunosuppressants, new medications, and storage requirements.
Staff shortages and pharmacist rotation	Increase pharmacy staff members and maintain stable staffing within transplant care settings.
Fragmented communication and limited collaboration with physicians	Establish direct communication channels with physicians and improve multidisciplinary coordination through regular meetings.
Lack of structured assessment of patient understanding.	Implement approaches for assessing patient understanding, like the teach-back method, and use standardized counseling checklists.
Medication shortages	Improve medication supply estimation by using updated patient statistics and continuous stock monitoring
Fragmented dispensing systems	Develop a simple unified electronic registry linking dispensing points to prevent duplicate dispensing and improve patient tracking.

## 4. Discussion

### 4.1. Expanding yet constrained pharmacist role

Pharmacists are increasingly recognized as an integral part of the transplant team, as their role has expanded from traditional dispensing duties to encompass multiple distinct clinical roles. Previous studies have demonstrated that clinical pharmacy involvement in multidisciplinary teams and medication management has improved medication safety and reduced preventable side effects [8]. These expanding responsibilities can range from drug monitoring and identifying drug-drug and drug-disease interactions to managing toxicities and medication management, including changing, substituting, and even ordering laboratory tests to ensure medication safety and effectiveness [4].

However, pharmacists in our study described predominantly dispensing-centered responsibilities, with only a glimpse of evolving, more proactive behaviors. Notably, these evolving, proactive behaviors appeared more evident among senior pharmacists and in those with long continuity in the transplant setting, as they described more advanced clinical and administrative practices, which could imply that practical experience in the transplant field supports role development.

These findings suggest that the transition from traditional dispensing roles to advanced clinical roles remains incomplete. Participants consistently linked this not to lack of willingness, but to organizational barriers including administrative workload, frequent pharmacist rotation, and limited structured training.

In terms of administrative duties, such as file archiving, these duties were perceived as a work burden by participants; participants suggested that the transition of selected administrative duties to non-pharmacist staff would allow pharmacists to focus on their clinical roles.

On the other hand, the lack of structured pharmacist training increased reliance on practical experience for role development in the transplant setting and was perceived as a contributing factor to role differences between more and less experienced pharmacists. Furthermore, frequent pharmacist rotation between wards or pharmacy departments was described as affecting the cumulative practical experience gained through exposure to the transplant setting.

Taken together, these observations suggest that reducing routine administrative duties, maintaining greater workforce stability, and providing structured transplant-specific training may facilitate the advancement of pharmacists’ roles and their integration into multidisciplinary transplant care.

Similar limitations to pharmacists’ roles have also been reported in other studies conducted in China and in Tanzania. Both the Chinese and Tanzanian studies identified staff shortages, a lack of ongoing pharmaceutical training in health care facilities, and reduced time for pharmaceutical care provision due to assigning non-pharmaceutical responsibilities to pharmacists as the main barriers to improving clinical pharmacy services [9,10]. Similarly, other Iraqi studies conducted in different settings also highlighted similar concerns regarding pharmacists’ preparedness and the need for structured training to improve pharmacists’ professional roles [11].

This suggests that the perceived barriers by pharmacists in our study are not unique to transplant care and may reflect broader workforce and training challenges across different pharmacy practice settings. Furthermore, these findings suggest that workforce stability and structured professional development represent potential practical strategies to expand pharmacists’ clinical roles across comparable healthcare settings. However, the feasibility and effectiveness of these strategies would need to be evaluated in practice before they are recommended for wider implementation.

### 4.2. Fragmented communication and coordination

Interprofessional communication is considered essential for safe and coordinated transplant care. Previous multidisciplinary intervention studies demonstrated that pharmacists’ involvement in transplant patient medication management, like medication reconciliation and regular follow-ups, has resulted in reduced medication discrepancies, increased patient adherence, diminished acute infection and rejection, and thereby improved overall clinical outcomes [12].

In the current study, pharmacists reported different patterns of communication across transplant settings, which appears to reflect pharmacists’ role within their respective practice setting. Outpatient pharmacists generally reported more direct and effective communication with physicians due to the nature of pharmacists’ responsibilities within the consultation clinic, which requires coordination regarding prescription refills, medication availability, and patient follow-up issues, in addition to agreements regarding medication dispensing strategies, handling near-expiration-date medications, and medication need estimation reports. These activities often require constant and direct communication with the physician.

In contrast, inpatient pharmacists described fragmented collaboration with the physician and consistent daily collaboration with the nursing staff, which usually acts as an intermediary. This pattern of communication may reflect the limited clinical responsibilities described by inpatient pharmacists, which limits the opportunities for clinical interaction between pharmacists and physicians. Participants also linked this limited interaction to organizational factors, such as the absence of fixed ward rounds and pharmacists’ multi-ward responsibilities, as well as limited physician presence in the ward. On the other hand, effective inpatient pharmacist-nursing staff communication and reliance on them as intermediaries were linked to their continuous presence in the ward and their central role in coordinating day-to-day patient care activities.

Collectively, these findings suggest that communication patterns were strongly influenced by the organizational structure of care, positioning nurses as the communication bridge between pharmacists and physicians.

The current findings partially align with a previous Iraqi study suggesting that pharmacist-physician collaboration is influenced by contextual and practice-related factors, including practice setting, role specification, and interpersonal relationships [13].

In this study, other organizational and physical factors appear to substantially shape interprofessional communication. For instance, Pharmacists working in close proximity to the physician clinics reported more direct communication. In contrast, pharmacists working in distant locations perceived this as a major barrier to communication and had to rely on phone calls, WhatsApp groups, and monthly meetings to maintain coordination and continuity of care.

Although most participants perceived these adaptive strategies as practical solutions to overcome limited physician availability and distant working locations, one pharmacist expressed concerns about using them as a substitute for direct interaction, as these methods depend on physician availability and responsiveness, which could delay the decision-making process. Although this concern was raised by only one pharmacist, it demonstrates the possible fragility of these methods when relying on them and highlights the importance of implementing a formal communication pathway and considering these adaptive strategies only as a complement to the formal one.

Additionally, Interpersonal dynamics appear to influence the collaboration quality as well. Mutual trust, prompt physician responsiveness, and team spirit appear to facilitate communication and strengthen collaboration. In contrast, hierarchical attitudes and age differences occasionally contributed to resistance toward pharmacists’ recommendations, particularly among junior pharmacists.

Studies conducted in Iraq, Tanzania, and Brazil have also reported similar communication-related barriers. The Iraqi study reported inconsistent pharmacist-physician collaboration and variable acceptance of pharmacist recommendations depending on the individual physician and the practice setting [14]. Similarly, the Tanzanian study identified ineffective collaboration, hierarchical attitudes, and negative physicians’ perceptions of pharmacists, which resulted in difficulty integrating pharmacists into the multidisciplinary team [10]. Likewise, a Brazilian study highlighted limited understanding of the pharmacist’s clinical role and weak pharmacists’ training as barriers to integration [15].

Collectively, these findings suggest that communication and collaboration within the transplant setting are influenced not only by organizational factors but also by physical proximity, interpersonal relationships, healthcare providers’ hierarchy, and their ability to adapt strategies to overcome obstacles to collaboration. Consequently, addressing these communication gaps requires system-level interventions that support regular multidisciplinary interaction and more structured communication pathways.

### 4.3. Patient education and counseling practices

Kidney transplant recipients usually require a complex medication regimen, especially regarding immunosuppressant therapy, which makes patient understanding of their medication and proper adherence an essential component to the success of the transplantation process, as inadequate adherence may negatively affect the graft and might lead to transplant complications and rejection [16].

Accordingly, patient education has become an integral component of the pharmacist’s role; previous studies highlighted the pharmacist’s duty in increasing patients’ knowledge about their medication through proper counseling sessions and regular follow-ups [17]. These pharmacist-led educational interventions have also resulted in higher adherence rates, ultimately leading to better transplant outcomes [18].

In the current study, both inpatient and outpatient pharmacists described being involved in the patient educational process. However, their educational practices appeared to vary in content and in the counseling and reinforcement approaches.

In general, transplant education was medication-centered, which appeared consistent with previous literature, where pharmacists’ educational role focused mainly on medication use, side effects, and adherence, in addition to counseling related to therapy optimization [4].

However, few pharmacists described covering broader educational topics, beyond medication-related information. This variation in educational content, along with differences in delivery approaches, suggests a lack of a standardized educational framework, where patient education relies largely on initiative and proactive behaviors.

Some Participants described their role as enforcing the physicians’ instructions, which may reflect the ongoing educational needs of kidney transplant recipients, who require lifelong medication management and frequent treatment adjustments. This practice appears to be consistent with previous studies that recommend repeated and ongoing patient education sessions due to the nature of transplant education, which includes a large amount of complex information that is difficult for the patients to absorb in a single session. Previous literature also recommends a more structured educational approach that extends across the pre-, peri-, and post-transplant periods to prepare patients for lifelong medication management and encourage realistic expectations for post-transplant care [19].

Despite these recommendations, only a few pharmacists in this study reported using assessment methods like teach-back to assess patients’ understanding of the provided instructions. Nevertheless, previous studies have focused on such practices, as they have proven to improve patients’ understanding and self-management skills [20]. Participants attributed the limited use of assessment to the heavy workload, concerns about patient dissatisfaction caused by delays in patient flow, and the perception that assessing patient understanding was not part of their routine responsibilities.

In addition, some pharmacists reported involving caregivers in counseling sessions to reinforce medication-related information. This practice is consistent with previous studies, which highlight the importance of social support and caregiver involvement in improving patient adherence and supporting lifelong immunosuppressant therapy [21].

Collectively, these findings suggest that transplant education extends far beyond giving medication instructions, as repeated reinforcement, assessment of patient understanding, and caregiver involvement all contribute to the success of the educational process, ultimately leading to better patient adherence and clinical outcomes.

### 4.4. Medication management and system-related barriers

Medication access is considered a cornerstone to the success of the transplantation journey, as any interruption in immunosuppressant medication can negatively affect graft survival and patient outcomes [16].

Medication shortages appeared to be a worldwide challenge that is not limited to low-resource settings; shortages of immunosuppressants have been reported internationally and highlighted by transplant organizations [22].

In the current study, participants reported several supply and dispensing-related challenges that were linked to several system-related factors:

1. Inconsistent medication supply, where the received quantities were reported to be less than the requested ones, resulting in medication supply lower than the patient’s needs.
2. Outdated need estimation reports, prepared up to two years in advance, limiting their usefulness as an accurate predictor of the current need.
3. Increasing medication demand resulting from the natural growth in the registered transplant recipients’ number, in addition to patient redistribution from their originally assigned center to the current center, which adds to the recipients’ number and exacerbates the already existing supply challenges.

These factors are consistent with an Iraqi study reporting system-related challenges affecting need estimation and medication supply processes [23], and with broader reports of procurement and supply chain challenges that affect the timely availability of essential medications in Iraq [24].

Despite these challenges, pharmacists reported several approaches to overcome supply challenges, such as medication redistribution between hospitals and readjusting the dispensed quantity per patient, in addition to keeping an emergency stock for admitted patients. These approaches can be described as a temporary solution aimed at maximizing medication availability during shortages. On the other hand, some participants reported a more sustainable solution through improving the medication estimation process using updated monthly and yearly statistics.

These actions reflect pharmacists’ efforts to ensure continuity of care despite these limitations and highlight pharmacists’ perceptions of the critical nature of these medications and the catastrophic results if they were discontinued.

Likewise, adaptive strategies have also been recognized in the previous literature as mitigation strategies used during medication shortages [25].

Beyond system- and dispensing-related challenges, pharmacists also reported frequent changes in medication manufacturers, linking them to several practical and clinical challenges. These changes were described as a possible cause of healthcare providers’ confusion due to administration and preparation differences, in addition to causing patient confusion and uncertainty.

Similar concerns were also reported in previous studies, where medication shortages and switching between formulations led to patient confusion, medication errors, and fluctuating blood drug levels, particularly when non-equivalent formulations were used [26].

These findings suggest that medication shortages may create additional challenges beyond those related to medication unavailability, especially in transplant settings, as immunosuppressant medication requires careful handling.

Beyond medication shortages, pharmacists also described additional challenges related to fragmented follow-up care and disconnection between dispensing points, which were perceived as limiting patient information exchange and allowing for possible duplicate dispensing, collectively adding further difficulties to the medication management process. Furthermore, geographical and financial barriers may indirectly contribute to medication management problems by limiting updates to patients’ follow-up information.

Comparable findings have been reported in previous studies. A Colombian study highlighted the negative impact of a fragmented healthcare system after transplantation, where increasing the number of healthcare providers involved in patient care was associated with a higher risk of mortality [27].

Overall, these findings reflect the complex interaction between supply-chain problems and healthcare-related challenges. Although medication shortages were the central challenge, their impact appeared to generate a cascade of secondary challenges, such as frequent manufacturer changes, duplicate dispensing, fragmented follow-up, and information-sharing challenges, reflecting the complexity of the medication management process. This also emphasizes the need for approaches that address both medication availability and continuity of care.

When the four themes are considered together, a broader picture emerges, in which transplant care is maintained through pharmacists’ initiatives and adaptation, in an attempt to compensate for organizational and system-level constraints. However, these adaptive roles and strategies, such as reviewing prescriptions, improving need estimation reports, providing reports to the pharmacovigilance center, using informal education and communication strategies, and methods to handle drug shortages, all of them helped to maintain day-to-day care delivery. But they could not compensate for the absence of more structured and integrated systems of care.

## 5. Limitations

This study has several limitations. First, the participants were recruited from only two hospitals in Baghdad, which may limit the transferability of the study results to other Iraqi settings. Second, the study explored pharmacists’ perspectives only, apart from physicians’ and patients’ perspectives, which limited opportunities for triangulation across stakeholder groups. Additionally, as the interviews relied on self-reported experience, the participants may have provided socially desirable responses, despite assurance of anonymization. Third, the participant responses were translated from Arabic to English for coding and analysis; the translation process may have resulted in some loss of contextual nuance despite researchers’ efforts to preserve the data’s meaning. Finally, the first author was a pharmacist and a master’s candidate; this background may have influenced the data collection process and interpretation despite measures taken to enhance reflexivity and analytical rigor.

## 6. Conclusions

This study indicated that pharmacists in governmental transplant units play an evolving role in caring for transplant patients. Despite system-related and workflow challenges, pharmacists demonstrated adaptive approaches in an attempt to maintain medication access and patient support with the available resources.

However, these findings also highlight challenges that extend beyond the capacity of individual pharmacists to resolve, for instance, limited clinical roles, communication gaps, fragmented follow-up, and medication supply issues.

These findings highlight the need for more structured organizational support, clearer communication pathways, and an integrated system to strengthen Iraqi transplant care practices within comparable governmental settings.

## Supporting information

S1 File Arabic version of the pharmacist`s interview guide. (DOCX)

S2 File English version of the pharmacist`s interview guide. (DOCX)

S3 File Illustrative analytical structure linking final themes, subthemes, example codes, and representative quotations. (DOCX)
