# Supplementary material for: Barriers and enablers shaping kidney transplant care delivery: Insights from Iraqi pharmacists
Source: PLoS One. 2026 Oct 12;21(10):e0360647. doi: 10.1371/journal.pone.0360647 (PMC13657322; doi:10.1371/journal.pone.0360647)
Supplement: Supplementary file 1 [file pone.0360647.s001.docx]

**مقابلة الصيدلي**

القسم الاول: المعلومات الأساسية

• التخصص / مكان العمل:

• عدد سنوات خبرتك في رعاية مرضى زراعة الكلى:

• المستشفى / مكان العمل:

القسم الثاني: الأسئلة الرئيسية

1. نطاق الدور والمسؤوليات

- هل يمكن ان تشرح لي مسؤولياتك تجاه مرضى زراعة الكلى؟ كيف يبدأ دورك عادة مع المريض,و ما الخطوات او المهام التي تقوم بها اثناء المتابعة اليومية او الدورية؟

2. الدور التعليمي

- هل تقدم تثقيفاً للمرضى بعد الزراعة؟ وكيف يتم ذلك عادةً (كلام مباشر، كتيّب، وسائل أخرى)؟ وما المواضيع التي تغطيها؟ وهل تستخدم طريقة معينة لضمان استيعاب المريض لهذه المعلومات؟
- ما أبرز الصعوبات اللي تواجهها أثناء تقديم التثقيف للمرضى؟
- هل تلقيت اي دورات او برامج بخصوص رعاية مرضى زراعة الكلى تؤهلك للقيام بهذا الدور؟

3. التعاون وتبادل المعلومات

- صف لي آلية التواصل والتعاون مع باقي مقدمي الرعاية الصحية (الأطباء، الممرضين، الصيادلة) في رعاية مرضى زراعة الكلى؟
- برأيك, ما العوامل التي تساعد على نجاح هذا التعاون؟
- ما التحديات التي تواجهك بخصوص موضوع التواصل أو توثيق المعلومات؟

4. توفر الأدوية والوصول إلها

- من خلال خبرتك، ما الصعوبات التي يواجهها المريض في الحصول على أدويته بعد الزراعة؟
- كيف تتعامل إذا حدث نقص في الأدوية، أو احتجت الى استبدالها ؟

5. التحديات في عملك

- ما أبرز التحديات أو المعوّقات التي تواجهك أثناء تقديم خدمات الصيدلة لمرضى زراعة الكلى؟

6. مقترحات للتطوير

- برأيك, ما الجوانب الايجابية في النظام الحالي التي اثبتت فعاليتها والتي يجب الحفاظ عليها؟
- وما التغييرات أو التدخلات التي يمكن ان تحسّن خدمات الصيدلة المقدمة لمرضى زراعة الكلية؟

7. خاص بصيدلي العيادة الخارجية

- عند التعامل مع مريض اجرى عملية زرع الكلى في مكان اخر (مركز خاص / محافظة اخرى / خارج البلد)، ما أبرز المشكلات التي تواجهك؟
