# Supplementary material for: Barriers and enablers shaping kidney transplant care delivery: Insights from Iraqi pharmacists
Source: PLoS One. 2026 Oct 12;21(10):e0360647. doi: 10.1371/journal.pone.0360647 (PMC13657322; doi:10.1371/journal.pone.0360647)
Supplement: Supplementary file 2 [file pone.0360647.s002.docx]

**Pharmacist interview**

**Section A: Basic Information**

• Profession/Position:

• Years of experience in transplant care:

• hospital/ place of work:

**Section B: Main Questions**

1. **Scope of role & responsibilities**

- Can you describe your main responsibilities toward kidney transplant patients? How does your role usually begin with the patient, and what tasks do you perform during daily or periodic follow-up?

**2. Educational role**

- Do you provide post-transplant education to patients? How is this usually provided (Verbally, through a booklet, or by other means)? What topics do you cover? Do you use any particular method to ensure patients' understanding of the information provided?
- What difficulties do you face when providing education?
- Have you received any training courses or programs related to kidney transplant care that prepare you for this role?

**3. Collaboration and information sharing**

- Can you describe how communication and collaboration with other healthcare providers work?
- What helps this collaboration succeed?
- What problems do you face in communication or documentation?

**4. Medication availability and access**

- From your experience, what difficulties do patients face in obtaining their medications after transplantation?
- How do you manage medication shortages or situations in which substitution is required?

**5. Barriers in your work**

- What are the main challenges you face in delivering pharmacy services for transplant patients?

**6. Suggestions for improvement**

- What practices are already working well and should be continued?
- What changes or interventions would improve pharmacy services for transplant patients?

**7. (Outpatient pharmacists only)**

- When dealing with patients who underwent kidney transplantation elsewhere (private center/ other province/ abroad), what main challenges do you encounter?
