# Supplementary material for: Barriers and enablers shaping kidney transplant care delivery: Insights from Iraqi pharmacists
Source: PLoS One. 2026 Oct 12;21(10):e0360647. doi: 10.1371/journal.pone.0360647 (PMC13657322; doi:10.1371/journal.pone.0360647)
Supplement: Supplementary file 3 [file pone.0360647.s003.docx]

**Table S1. Illustrative analytical structure linking final themes, subthemes, example codes, and representative quotations.**

| **Final theme** | **subtheme** | **Example codes** | **Representative quotation** |
| --- | --- | --- | --- |
| 1. Dispensing-centered practice within a constrained and evolving pharmacist role. | Dispensing and administrative tasks as the dominant professional identity. | Medication supply management and dispensing planning | Ph6: “Our unit monitors available medication quantities within the hospital and provides guidance to physicians and outpatient pharmacists regarding the monthly dispensing strategy.” |
|  |  | Restricted clinical role and authority | Ph12: “It is possible to review laboratory results and ask questions or make comments; however, this is usually done for personal learning and practical benefit, rather than as formal recommendations to physicians.” |
|  | Emergence of informal and experience-driven practice in the context of limited training. | Advanced pharmacists' roles. | Ph7: “When the same complaint is reported by several patients, this indicates that there may be a real problem.  In such cases, we report the issue to the pharmacovigilance committee in the hospital |
|  |  | Pharmacy staff rotation limits role development | Ph3: “The staff rotation system leads to frequent changes in personnel. Sometimes, before a pharmacist fully acquires all necessary expertise, they are transferred to another department.” |
| 2. Fragmented yet adaptive interprofessional collaboration | Variation in collaboration patterns across practice settings. | Pharmacist-physician collaboration | Ph13: “As an outpatient pharmacist, our communication is primarily with physicians only, and we do not have direct interaction with the nursing staff. |
|  | Indirect and mediated communication with physicians as a structural barrier | Limited and indirect communication with physicians | Ph11: “I believe that the main challenge we face is the lack of communication with physicians” |
|  | Use of informal and digital communication strategies as practical workarounds | Use of informal and digital communication pathways | Ph4: “Through WhatsApp group, we can quickly inform each other about available medications, discuss any issues, and agree on how medications will be dispensed. |
|  | Collaboration enabled by interpersonal trust, organizational coordination, and physical proximity. | Interpersonal dynamics facilitate collaboration | Ph8: “This collaboration can be described as very friendly and smooth, as we work here as one team, which contributes to the success of the collaboration.” |
| 3. Patient education as an informal and inconsistently delivered practice | Limited, medication-focused and non-standardized patient education | Nursing staff is responsible for patient education | Ph1: “ Communication is primarily with the nursing staff. Therefore, the educational role of pharmacists can be described as indirect” |
|  |  | Providing medication instructions to patients | Ph2: “Patient education is provided during routine visits. This typically includes counseling on proper medication use and potential side effects that may occur.” |
|  | Variable patient engagement and limited assessment of understanding | Lack of assessment of patient understanding | Ph10: “ I don’t use an assessment method; I just provide the instructions, and then I follow up by checking the remaining medications to ensure that the treatment has been taken. |
|  |  | Involving caregiver in patient education | Ph5: “ In many cases, education is delivered to family members rather than directly to the patient.” |
| 4. Medication availability within a constrained and variably regulated supply system | Predominantly stable availability with episodic and external shortages | Temporary shortages | Ph8: “ Regarding medications, most drugs are available—we can say around 80%. However, shortages occur from time to time.” |
|  | System-level drivers undermining medication supply stability | Outdated need estimation reports leading to shortages | Ph7: “Currently, medication needs are estimated two years in advance. For example, in 2025 we estimate the medication needs for 2027. This approach is problematic because the estimation may become inaccurate. |
|  | Adaptive yet constrained responses to medication shortages | Adjusted medication supply due to shortages | Ph13: “Sometimes we also divide the available medication quantities among patients, so that each patient receives part of their monthly supply instead of the full quantity, in order to ensure that all patients receive at least some treatment, in accordance with the principle of equity.” |
|  | Product variability as a source of patient uncertainty | Patient concern about drug product changes | Ph9: “Many Iraqi patients tend to have limited trust in the healthcare system when such changes occur. As a result, they may become concerned about the new product and sometimes schedule an appointment with their physician in a private clinic simply to ask whether it is safe to take the new medication.” |
|  | Fragmented follow-up and disconnected medication management across care settings | Prescription inconsistencies and clinical discrepancies | Ph4: “One of the main challenges is receiving unclear prescriptions or dosages that appear inconsistent or illogical, and it is often difficult to communicate directly with their external physician to verify the information. |
