# Supplementary material for: Barriers and enablers shaping kidney transplant care delivery: Insights from Iraqi pharmacists
Source: PLoS One. 2026 Oct 12;21(10):e0360647. doi: 10.1371/journal.pone.0360647 (PMC13657322; doi:10.1371/journal.pone.0360647)
Supplement: Supplementary file 4 [file pone.0360647.s004.docx]

**Responses to Academic Editor Comments:**

| **Editor comments** | **Author response** |
| --- | --- |
| 1. Improve methodological transparency and qualitative rigor | Thank you for this valuable comment. We have revised the Methods section to improve methodological transparency and qualitative rigor. We expanded the description of participant recruitment, non-participation, interviewer reflexivity, interview setting, data saturation, transcription and translation procedures, coding and theme development, and ethical considerations. These revisions provide a more comprehensive description of the study procedures in accordance with the COREQ recommendations. |
| 2. Strengthen the reflexivity statement | In response to this comment. We expanded the reflexivity section by adding further information on the interviewer–participant relationship and the measures used to minimize interviewer and analytical bias. During the interviews, the first author periodically summarized and paraphrased participants’ responses to confirm understanding and seek clarification. In addition, the Arabic transcripts and their English translations were reviewed by the second author before coding, and coding decisions, theme development, and thematic refinement were discussed with the second author throughout the analysis until consensus on the final thematic structure was reached. The researcher’s professional background, which was already described in the original manuscript, was retained. |
| 3. Strengthen the interpretation of findings | Thank you for this valuable comment. The Discussion was revised to reduce repetition of the study findings and place greater emphasis on their interpretation. The possible reasons underlying the identified barriers and the relationships between them were further discussed, particularly in relation to organizational and health-system factors. Their potential implications for transplant care and multidisciplinary collaboration were also clarified where relevant. |
| 4. Expand engagement with relevant qualitative literature | Thank you for this important suggestion. The Discussion was revised to better integrate the existing literature with our findings rather than relying mainly on direct comparisons with previous studies. This allowed the findings to be discussed within a broader context, particularly in relation to organizational challenges, communication and collaboration, patient education, and medication-related system issues. |
| 5. Review reporting against the COREQ checklist | We appreciate the reviewer for highlighting the importance of comprehensive reporting according to the COREQ checklist. We carefully reviewed the manuscript against the COREQ guideline and revised the Methods section accordingly. All applicable COREQ items relevant to our study have now been addressed. As recommended, we clarified the audio-recording, transcription, translation, and verification process, as well as the procedures used to enhance the trustworthiness of the analysis. Items that were not undertaken (e.g., participant transcript verification/member checking, repeat interviews, and field notes) have not been reported to accurately reflect the study methods. |
| 6. Better justify the sampling strategy | We appreciate this valuable suggestion. We strengthened the justification for the sampling strategy by clarifying that all eligible pharmacists across the two participating transplant centers were invited to participate. The majority of eligible pharmacists participated, and information saturation was achieved, supporting the adequacy of the sample to address the study objectives. |
| 7. Additional comments | We thank the reviewer for these helpful additional comments. The manuscript has been revised accordingly.  * The Abstract has been revised accordingly.  * The Introduction has been strengthened by clarifying the rationale for selecting pharmacists as key informants and emphasizing the novelty of the study.  * The Methods section has been revised to address the reviewer’s comments.  * Participant quotations have been reduced where appropriate while maintaining adequate support for each theme.  * Figure 1 has been removed. Figure 2 has been retained because it provides an analytical synthesis of the findings.  * The Limitations section has been expanded to explicitly acknowledge potential social desirability bias and further clarify already listed ones.  * The Discussion was shortened, and language throughout the manuscript has been revised accordingly. |

**Responses to reviewer 1**

| **Reviewer comments** | **Author Responses** |
| --- | --- |
| 1.Insufficient reporting of qualitative rigor | We appreciate the reviewer’s valuable comments. To address these concerns, we expanded the description of the qualitative analysis by clarifying how information saturation was determined, describing how differences in coding and interpretation were resolved through discussion until consensus was reached, and explaining that the coding framework and themes were iteratively reviewed and refined throughout the analysis until the final thematic structure was established. |
| 2. Limited reflexivity | In response to the reviewer’s valuable suggestion, we expanded the reflexivity section by clarifying the interviewer–participant relationship and describing how the interviewer’s professional background influenced participant rapport. We also added further details on the measures used to minimize potential interviewer and analytical bias during data collection and analysis, including the use of open-ended interviews, clarification of participants’ responses, and discussion of code and theme development with the second author throughout the analysis process. |
| 3. Discussion is excessively descriptive | We appreciate this important observation. We carefully revised and shortened the Discussion, particularly the sections that repeated findings already presented in the Results. We paid more attention to explaining the findings, the possible reasons behind the reported barriers, and how some of these challenges may lead to or worsen others. We also discussed their practical consequences for transplant care where relevant. |
| 4. Greater engagement with qualitative literature | Thank you for highlighting this point. We revised the Discussion to make better use of the available literature when interpreting our findings, rather than simply comparing whether similar findings had been previously reported. We used relevant evidence to provide further context for the challenges identified in this study and to support their interpretation within the healthcare setting. |
| 5. COREQ reporting | In response to this comment, we carefully reviewed the manuscript against the COREQ checklist and revised the Methods section accordingly. Additional details regarding the interviewer–participant relationship, non-participation, information saturation, and other relevant COREQ items have been incorporated where applicable. |
| 6. Sampling justification | In response to this comment, we further justified the sampling strategy by clarifying that all eligible pharmacists from the only two governmental kidney transplant centers in Baghdad were invited to participate. The majority of eligible pharmacists participated, and information saturation was achieved, supporting the adequacy of the sample for the study objectives. |
| 7. Minor comments | Thank you for these helpful comments. The manuscript was revised accordingly. The requested information was added to the Abstract, Introduction, and Methods. The Results and Discussion were shortened to reduce repetition, and Figure 1 was removed while Figure 2 was retained. The Limitations section was expanded as suggested, and the manuscript was carefully revised for language and consistency. |
