# Supplementary material for: Barriers and enablers shaping kidney transplant care delivery: Insights from Iraqi pharmacists
Source: PLoS One. 2026 Oct 12;21(10):e0360647. doi: 10.1371/journal.pone.0360647 (PMC13657322; doi:10.1371/journal.pone.0360647)
Supplement: Supplementary file 5 [file pone.0360647.s005.docx]

|  | **Reviewer comments** | **Author responses** |
| --- | --- | --- |
| **1.** | The revised Methods should clearly explain:  *The overall qualitative methodology and epistemological or theoretical orientation. *Whether coding was inductive, deductive, or a combination of both. *Whether the analysis focused on explicit meanings or underlying interpretations. *How initial codes were grouped, reviewed, and transformed into themes. *Whether a coding framework or codebook was developed and modified. *How differences between the two analysts were handled in practice. *How the authors determined that the final themes represented patterns of shared meaning rather than broad topic categories.  *PLOS ONE requires qualitative analytical methods to be explicit, systematic, and sufficiently detailed, and recommends reporting according to COREQ or SRQR. | Thank you for this important comment. We have revised the Methods section to provide a clearer description of the study methodology and analytical approach.  The study is now identified as using a qualitative descriptive approach, selected to provide a direct description of pharmacists’ perspectives and experiences. We have also clarified that Braun and Clarke’s reflexive thematic analysis was used.  The analysis was primarily inductive, with codes generated from the collected data rather than from a predefined coding framework, and was conducted at a semantic level, focusing on the explicit meanings reported by participants rather than latent meanings.  Although the broad areas of analysis were guided by the interview guide, the final theme names and content, as well as the subthemes, were developed from the collected data.  We also expanded the Methods section to describe the analytical process more clearly. We also clarified the role of the second author.  *COREQ is provided as a supplementary file |
| **2.** | The interview guide covered pharmacists’ responsibilities, patient education, interprofessional collaboration, medication availability, barriers, and recommendations. The four final themes were pharmacist role, communication and collaboration, patient education, and medication availability.  Clarify whether the interview-guide domains formed an initial deductive coding structure. | We appreciate this valuable notice. We clarified that although the broad areas of analysis were guided by the interview guide, they did not form a predefined deductive coding structure. Initial codes were generated inductively from the collected data, while the final theme names, content and subthemes were developed through the analysis. |
|  | Provide a coding tree or table showing the progression from quotations to codes, subthemes, and final themes. | Thank you for this suggestion. We have added a supplementary analytical table showing the progression from representative quotations to example codes, subthemes, and final themes. As no formal coding tree was developed during the analysis, the table is presented as an illustrative analytical structure rather than a retrospective codebook. |
|  | Explain which findings emerged unexpectedly from the interviews | We agree that distinguishing findings that arose from the analysis from the areas anticipated in the interview guide would improve transparency.  We clarified this point at the beginning of the Results section, noting that while the themes remained related to the areas the study intended to explore, the analysis also generated additional findings not predefined by the interview guide. |
|  | Describe any contradictory, negative, or deviant cases. | Thank you for this important comment. We reviewed the Results to ensure that contrasting, less commonly reported, and differing perspectives were adequately represented rather than limiting the findings to the dominant views. Across themes, we retained and reported variation in participants’ experiences and practices where present.  Within the pharmacist-role theme, we presented the dominant dispensing- and administrative-focused role alongside the experiences of pharmacists who described undertaking additional activities beyond their formally assigned responsibilities.  In the collaboration theme, we explicitly described differences between inpatient and outpatient pharmacists and between the two hospitals. The findings also included both facilitating and challenging aspects of communication, as well as perspectives reported by only a small number of participants. To further strengthen the representation of such differing perspectives, we added the concern expressed by one pharmacist regarding overreliance on informal communication methods, despite these methods being described by other participants as useful adaptive strategies.  We also retained variation within the patient-education theme. Although most pharmacists described providing patient education, the content and method of education were not consistent across participants. Some pharmacists provided broader education beyond medication-related information, while educational delivery varied between verbal counseling, written instructions, and indirect education through nursing staff. Similarly, not all inpatient pharmacists reported discharge counseling, further illustrating variation in practice rather than a uniform approach.  For medication availability and access, the findings did not reveal directly opposing views; instead, participants described different problems according to their individual experiences and practice settings. For example, outpatient experiences included patient concerns or reluctance when medication manufacturers changed, whereas inpatient experiences highlighted potential confusion among healthcare staff arising from differences between available products. We therefore retained these different experiences rather than presenting an artificial contradiction when the data did not support it. |
|  | Show more clearly how experiences differed between inpatient and outpatient pharmacists, between the two hospitals, and between less and more experienced pharmacists. | Thank you for this important comment. We reviewed the Results to ensure that differences between inpatient and outpatient pharmacists and between the two hospitals were clearly represented, and these were already reflected across the relevant themes.  However, differences related to pharmacists’ level of experience were less explicit. Therefore, we revised the pharmacist-role theme to highlight that several evolving practices were described by more experienced pharmacists. This pattern is also further interpreted in the Discussion in relation to accumulated experience and stable staffing. |
|  | Consider whether the current four “descriptive themes” would be more accurately described as categories with analytical subthemes. | Thank you for this helpful suggestion. We considered whether the four overarching findings would be more appropriately described as categories. However, after reviewing the thematic structure, we retained the term “themes,” as each represents a recurring pattern across participants’ accounts rather than only a broad topic corresponding to the interview-guide domains.  Although the broad areas were anticipated in the interview guide, the final theme names and content reflect the patterns identified through the analysis. The revisions made throughout the Results were also intended to make these patterns and the variation within each theme more explicit. |
|  | The manuscript would benefit from a stronger overarching interpretation | Thank you for this helpful suggestion. We agree that a stronger overarching interpretation would improve the integration of the study findings. Therefore, we added a concluding paragraph to the Discussion that brings the four themes together and highlights the broader pattern across them. |
| **3.** | Please clarify: *Whether saturation was assessed prospectively during data collection or retrospectively after all interviews. *After which interview no new codes were identified. *Who assessed saturation and how it was documented. *Whether the authors mean code saturation, meaning saturation, or general informational sufficiency.  *Whether saturation was assessed separately for inpatient and outpatient pharmacists. *Whether the three non-participants differed meaningfully from participants in practice setting or experience. | Thank you for this important clarification. We agree that recruitment in this study is more accurately described as total-population sampling rather than as being determined by saturation. Therefore, recruitment ended because all available eligible pharmacists had been interviewed, rather than because a predetermined saturation threshold had been reached.  We have revised the Methods accordingly and removed the previous statement regarding saturation.  Regarding the three non-participants, two were outpatient pharmacists, and one was an inpatient pharmacist. The two outpatient pharmacists were not senior staff, and pharmacists with comparable practice settings and experience were represented among the participants.  Although the non-participating inpatient pharmacist held a more senior role, three other inpatient pharmacists from the same hospital participated in the study, including pharmacists with substantial experience. |
|  | The sample also included pharmacists with only one month of transplant experience, while the maximum experience was six years. The authors should justify why one month was considered sufficient exposure and discuss whether the accounts of recently appointed pharmacists differed from those of experienced pharmacists. | Thank you for raising this point. No minimum duration of transplant experience was specified because the study aimed to reflect pharmacists’ perspectives and experiences as they existed in routine practice.  Pharmacists with limited experience were assigned to the transplant service and had direct contact with transplant patients. In fact, they were the only inpatient pharmacists available in that hospital during the study period. Therefore, excluding them solely based on duration of experience would have excluded an important aspect of the actual practice setting.  Their inclusion also provided insight into the experiences of recently appointed pharmacists, including challenges related to limited practical experience. |
| **4.** | *The study setting is central to the findings because the authors repeatedly attribute differences in communication and collaboration to whether a hospital had a dedicated transplant centre, the physical proximity of professionals, and the organisation of workflow. However, the Methods provide little information about the participating services beyond describing them as two public hospitals in Baghdad.  *The assertion that these are the “only two governmental hospitals” providing the relevant services in Baghdad should also be supported by an appropriate official or institutional source. | Thank you for this comment. We expanded the Study Setting section to provide further contextual information about the two hospitals.  We also clarified the important differences in outpatient service organization between the hospitals, particularly regarding dedicated transplant facilities, physician availability, pharmacy organization, and the physical proximity of services. These contextual details provide additional background for interpreting the differences between the two settings identified in the findings.  Regarding the statement that these were the only two governmental hospitals providing kidney transplant services in Baghdad, we were unable to identify an official published source that could verify this information, as it was based on verbal information available to the researchers. Therefore, to avoid making an unsupported claim, we revised the wording from “the only two” to “two major.” |
|  |  |  |
| **5.** | *The authors state that Arabic transcripts were translated into English using an “AI-assisted translation tool,” reviewed against the original Arabic by the first author, and subsequently reviewed by the second author before coding. This is currently insufficient from both methodological and research-ethics perspectives.  *PLOS requires authors to disclose the names of AI tools, how the tools were used, how their outputs were validated, and which parts of the study or data were affected. | *Thank you for this important comment. We have revised the Methods to provide further details regarding the AI-assisted translation process.  The interviews were manually transcribed verbatim and de-identified by the first author before translation. ChatGPT (OpenAI) was used only as an assistive translation tool, and successive de-identified transcript excerpts were entered rather than complete transcript files. No participant names, hospital identities, or other identifying information were submitted to the tool.  *The first author, an MSc candidate in Pharmacy with good English proficiency (IELTS overall score 7.5), reviewed each translated section against the original Arabic transcript and corrected translation errors, context-specific expressions, and terminology where necessary. The Arabic and English transcripts were subsequently reviewed by the second author before coding.  *Coding was conducted using the English-translated transcripts. The quotations reported in the manuscript were taken directly from these translations and were not translated separately for publication.  *ChatGPT is a cloud-based service. The specific model variant used at the time was not recorded; however, the translations were conducted using the GPT-5.2 family available at that time  *The ethics approval and consent did not specifically address the use of an external AI service; however, AI-assisted translation was performed only after complete de-identification, and no identifiable participant or institutional information was submitted. |
| **6.** | PLOS requires the minimal data necessary to support reported findings to be available. When human-participant data cannot be shared publicly for ethical or confidentiality reasons, the Data Availability Statement must explain the restriction and identify how qualified researchers may request access. | Thank you for this comment. We have revised the Data Availability Statement to clarify that all data that can be shared without compromising confidentiality are included in the manuscript and Supporting Information. The complete interview transcripts cannot be shared because of the risk of re-identification and because participant consent did not include permission for sharing interview data with other researchers. |
| **7.** | The manuscript includes reports of patients missing doses after manufacturer changes and a participant’s statement that differences between anti-thymocyte globulin products had “in some cases” resulted in dosing errors. The Discussion also refers to duplicated dispensing and “medication leakage,” although the Results provide limited supporting evidence for these claims.  The authors should clarify: *Whether these were documented incidents, direct observations, participant perceptions, or hypothetical risks. *How many participants reported each issue. *Whether dosing errors led to patient harm. *Whether these events were reported through formal pharmacovigilance or incident-reporting systems. *Whether “medication leakage” refers to duplicate dispensing, diversion, wastage, or another process. *Whether sufficient supporting quotations can be included without compromising confidentiality.  Throughout the Results and Discussion, wording should consistently indicate that these are pharmacists’ reported experiences or perceptions | Thank you for this important comment. We reviewed the relevant findings and revised both the Results and Discussion to more clearly distinguish between commonly reported experiences and isolated participant observations.  The quotations relating to ATG-related concentration differences and possible dosing errors, as well as the quotation describing possible missed doses following manufacturer changes, were already included in the manuscript.  We have now clarified the frequency of these findings in the Results, including that patient concern and confusion were reported by six pharmacists, while missed doses and dosing errors were each reported by individual participants.  We also added a supporting quotation for concerns regarding possible duplicate dispensing and clarified that this issue was raised by two pharmacists. The terminology was revised to avoid implying that duplicate dispensing was a formally documented event, and the previous term “medication leakage” was removed.  Finally, the Results and Discussion were revised for consistency, with more cautious wording throughout to indicate that these findings reflect pharmacists’ reported observations and perceptions. We could not determine from the available data whether the reported dosing or dispensing incidents resulted in patient harm or were formally documented; therefore, no such claims are made in the revised manuscript. |
| **8.** | The manuscript now states that the first author was a pharmacist and master’s candidate, had no prior professional or supervisory relationship with participants, and that professional similarity facilitated rapport. This is useful, but reflexivity should extend beyond stating that potential bias was “minimized.”  Please discuss: *The second author’s professional background and relationship to the research topic. *How the authors’ assumptions about Iraqi pharmacy practice may have affected questioning, coding, and interpretation. *Whether the interviewer’s status as a pharmacist encouraged openness or generated socially desirable professional accounts. *Whether reflexive notes, analytical memos, or an audit trail were maintained. *How interpretations that challenged the researchers’ initial assumptions were handled. *Whether field notes, repeat interviews, transcript return, or participant checking were undertaken. | Thank you for this comment. Several of the points raised had already been addressed in the previous revision. The manuscript already reports that the first author, who conducted the interviews, was a pharmacist and MSc candidate with no prior professional or supervisory relationship with participants.  It also describes the use of summarizing and paraphrasing during interviews to confirm understanding, the iterative review of interpretations during analysis, and acknowledges the potential influence of the first author’s professional background in the Limitations.  In the current revision, we have additionally clarified the role of the second author. He is an academic clinical pharmacist with a PhD in clinical Pharmacy and published more than 3 qualitative studies. He served as the research supervisor who supervised and revised every step. He was not involved in recruitment or interviewing, had no direct contact with participants during data collection, and was not introduced to them as part of the research team at that stage.  We also confirm that no field notes, repeat interviews, transcript return, formal participant checking, reflexive memos, or formal audit trail were undertaken. These procedures are therefore reported as not performed rather than being retrospectively inferred. |
| **9.** | *The study was limited to two hospitals in Baghdad and included pharmacists only. The authors themselves recognise that this limits transferability and prevents triangulation with physicians and patients. Nevertheless, the title refers broadly to “Iraqi pharmacists,” and the conclusion states that the findings indicate what is needed to strengthen “Iraqi transplant practices.”  The authors should avoid implying that the results represent Iraq nationally.  *The abstract and conclusion should similarly refer to “the two participating Baghdad transplant centres” or “comparable governmental transplant settings,” rather than the entire Iraqi transplant system. | Thank you for this important observation. We agree that the findings should not be interpreted as nationally representative, and we have therefore revised the wording in the Abstract and Conclusion to more clearly reflect the two participating governmental hospitals in Baghdad and to avoid extrapolating the findings to transplant practice across Iraq.  However, we respectfully retained the current title. The phrase “Iraqi pharmacists” is intended to describe the nationality and professional background of the participating pharmacists rather than to imply national representativeness. The geographical and institutional scope of the study is clearly specified in the Methods and Limitations, where the study is explicitly described as being conducted in two governmental hospitals in Baghdad. |
| **10.** | Although the Discussion has been shortened, it continues to repeat the Results and makes several interpretations that are more certain than the data permit  The Discussion should:  *Distinguish clearly between study findings, authors’ interpretations, and proposed policy responses. *Avoid causal expressions such as “led to” when the data consist of cross-sectional participant accounts. *Remove repeated descriptions already presented in the Results. *Give greater attention to interactions among the themes. *Discuss why informal adaptations may be helpful in the short term but potentially fragile or unsafe as substitutes for formal systems. *Present recommendations as suggestions requiring implementation testing, rather than established solutions. *Avoid extrapolating from pharmacist perspectives to patient outcomes without patient-level data. | Thank you for this important comment. We revised both the Results and Discussion to more clearly separate descriptive findings from interpretation.  Interpretive wording was reduced in the Results, while repeated descriptions were removed from the Discussion and replaced with more focused interpretation and linkage across themes.  We also removed or softened causal and overly certain statements that were not directly supported by the data, including claims regarding staffing, administrative duties, and comparisons with physicians and nurses.  Informal adaptations are now discussed as useful but potentially fragile substitutes for formal systems, and recommendations are presented as potential strategies whose feasibility and effectiveness require evaluation before wider implementation.  We also avoided extending pharmacists’ perspectives to patient outcomes where patient-level data were not available. |
