# Supplementary material for: Barriers and enablers shaping kidney transplant care delivery: Insights from Iraqi pharmacists
Source: PLoS One. 2026 Oct 12;21(10):e0360647. doi: 10.1371/journal.pone.0360647 (PMC13657322; doi:10.1371/journal.pone.0360647)
Supplement: Peer review history

# Peer review: Decision Letter 0

**DOI:** 10.1371/journal.pone.0360647.r001

**Authors:** Ali Haider Mohammed

21 Jul 2026

Please submit your revised manuscript Sep 19 2026 11:59PM. If you will need more time than this to complete your revisions, please reply to this message or contact the journal office at plosone@plos.org. When you're ready to submit your revision, log on to https://www.editorialmanager.com/pone/ and select the 'Submissions Needing Revision' folder to locate your manuscript file.

- A letter that responds to each point raised by the academic editor and reviewer(s). You should upload this letter as a separate file labeled 'Response to Reviewers'.
- A marked-up copy of your manuscript that highlights changes made to the original version. You should upload this as a separate file labeled 'Revised Manuscript with Track Changes'.
- An unmarked version of your revised paper without tracked changes. You should upload this as a separate file labeled 'Manuscript'.

If you would like to make changes to your financial disclosure, please include your updated statement in your cover letter. Guidelines for resubmitting your figure files are available below the reviewer comments at the end of this letter.

If applicable, we recommend that you deposit your laboratory protocols in protocols.io to enhance the reproducibility of your results. Protocols.io assigns your protocol its own identifier (DOI) so that it can be cited independently in the future. For instructions see: https://journals.plos.org/plosone/s/submission-guidelines#loc-laboratory-protocols. Additionally, PLOS ONE offers an option for publishing peer-reviewed Lab Protocol articles, which describe protocols hosted on protocols.io. Read more information on sharing protocols at https://plos.org/protocols?utm_medium=editorial-email&utm_source=authorletters&utm_campaign=protocols.

As the corresponding author, your ORCID iD is verified in the submission system and will appear in the published article. PLOS supports the use of ORCID, and we encourage all coauthors to register for an ORCID iD and use it as well. Please encourage your coauthors to verify their ORCID iD within the submission system before final acceptance, as unverified ORCID iDs will not appear in the published article. *Only*  the individual author can complete the verification step; PLOS staff *cannot*  verify ORCID iDs on behalf of authors.

We look forward to receiving your revised manuscript.

Kind regards,

Ali HM

Academic Editor

PLOS One

Journal Requirements:

When submitting your revision, we need you to address these additional requirements.

1. Please ensure that your manuscript meets PLOS ONE's style requirements, including those for file naming. The PLOS ONE style templates can be found at

https://journals.plos.org/plosone/s/file?id=wjVg/PLOSOne_formatting_sample_main_body.pdf and

https://journals.plos.org/plosone/s/file?id=ba62/PLOSOne_formatting_sample_title_authors_affiliations.pdf

2. When completing the data availability statement of the submission form, you indicated that you will make your data available on acceptance. We strongly recommend all authors decide on a data sharing plan before acceptance, as the process can be lengthy and hold up publication timelines. Please note that, though access restrictions are acceptable now, your entire data will need to be made freely accessible if your manuscript is accepted for publication. This policy applies to all data except where public deposition would breach compliance with the protocol approved by your research ethics board. If you are unable to adhere to our open data policy, please kindly revise your statement to explain your reasoning and we will seek the editor's input on an exemption. Please be assured that, once you have provided your new statement, the assessment of your exemption will not hold up the peer review process.

3. Please include a copy of Table 2.

4. If the reviewer comments include a recommendation to cite specific previously published works, please review and evaluate these publications to determine whether they are relevant and should be cited. There is no requirement to cite these works unless the editor has indicated otherwise.

Additional Editor Comments:

In preparing your revised manuscript, please address the following points carefully.

Major comments

1. Improve methodological transparency and qualitative rigor

Although the study employs Braun and Clarke's thematic analysis, several important components expected in qualitative research are insufficiently reported. Please provide greater detail regarding data saturation, reflexivity, transcript verification (member checking, if performed), maintenance of field notes or an audit trail, management of coding disagreements, and the evolution of the coding framework during analysis. These additions are important for establishing the credibility, dependability, and trustworthiness of the findings.

2. Strengthen the reflexivity statement

The manuscript currently provides only limited information regarding the interviewer. Please expand this section by discussing the researcher's background, relationship with participants, potential assumptions, and measures undertaken to minimise interviewer and analytical bias.

3. Strengthen the interpretation of findings

The Discussion currently remains largely descriptive and frequently reiterates the study findings before comparing them with previous literature. Please focus more on explaining why the identified barriers exist and discuss the findings within broader implementation, organisational, and health systems perspectives. Greater consideration of the implications for multidisciplinary collaboration and transferability to other low- and middle-income settings would considerably strengthen the manuscript.

4. Expand engagement with relevant qualitative literature

The interpretation would benefit from broader engagement with qualitative implementation research and health services literature, including organisational culture, implementation science, multidisciplinary collaboration, and health systems strengthening. Incorporating these perspectives would improve the depth of interpretation beyond comparisons with transplant pharmacy studies alone.

5. Review reporting against the COREQ checklist

Several reporting items recommended by the COREQ guideline remain insufficiently addressed. Please carefully verify the manuscript against the checklist, particularly regarding interviewer-participant relationship, repeat interviews, field notes, transcript verification, non-participation, and the process used to determine saturation.

6. Better justify the sampling strategy

Although the sample size is appropriate for qualitative inquiry, the rationale should extend beyond participant availability. Please provide a stronger justification for why the included participants were sufficient to address the research objectives.

Additional comments

Revise the Abstract to improve clarity and consistency. Please report the sampling strategy, specify the use of Braun and Clarke thematic analysis, indicate the number of participating hospitals, and correct sentence capitalization throughout.

Strengthen the Introduction by providing a clearer rationale for selecting pharmacists as key informants and by further emphasising the novelty and significance of the study.

Expand the Methods section by clarifying whether the interview guide was piloted, providing additional justification for verbal consent, describing the translation and verification process in greater detail, and reporting the software version used for qualitative analysis.

Consider reducing the number and length of participant quotations where similar concepts are repeatedly illustrated, while ensuring adequate support for each theme.

Reconsider the presentation of Figures 1 and 2. As both figures primarily provide conceptual summaries rather than analytical outputs, please evaluate whether both are necessary or whether a single figure would more effectively communicate the findings.

The Discussion may be shortened by removing repetitive descriptions of the results and placing greater emphasis on interpretation and practical implications.

Expand the Limitations section to acknowledge potential social desirability bias, limited transferability beyond Baghdad, the absence of physician and patient perspectives, and the possibility of translation-related bias.

Finally, please carefully revise the manuscript for grammatical accuracy, consistency of terminology, capitalization, tense usage, and overall presentation prior to resubmission.

[Note: HTML markup is below. Please do not edit.]

Reviewers' comments:

Reviewer's Responses to Questions

**Comments to the Author**

1. Is the manuscript technically sound, and do the data support the conclusions?

Reviewer #1: Partly

**********

2. Has the statistical analysis been performed appropriately and rigorously?

Reviewer #1: Yes

**********

3. Have the authors made all data underlying the findings in their manuscript fully available?

The PLOS Data policy

Reviewer #1: Yes

**********

4. Is the manuscript presented in an intelligible fashion and written in standard English?

Reviewer #1: Yes

**********

Reviewer #1: 1. Insufficient reporting of qualitative rigor

The manuscript states that Braun and Clarke thematic analysis was performed, but several essential components expected in qualitative research are missing or insufficiently described.

Please clarify:

whether data saturation was achieved and how this was determined;

whether transcripts were returned to participants for member checking;

whether reflexive notes or an audit trail were maintained;

whether coding disagreements occurred and how they were resolved;

whether the coding framework evolved during analysis.

These issues are particularly important for demonstrating trustworthiness and credibility.

2. Limited reflexivity

The manuscript briefly states that the interviewer was a pharmacist and master's student.

However, qualitative studies require a deeper discussion regarding:

prior relationship with participants

interviewer assumptions

possible influence on interviews

measures used to reduce interviewer bias

This section should be expanded.

3. Discussion is excessively descriptive

Most paragraphs simply repeat the findings before comparing them with previous literature.

The discussion would be stronger if it focused more on:

explaining why these barriers exist

linking findings to implementation science

discussing organizational behavior

considering health-system implications

discussing transferability to other LMICs

Currently the discussion resembles an extended Results section.

4. Greater engagement with qualitative literature

Most references discuss transplantation or pharmacy practice.

There is little discussion of:

qualitative implementation research

organizational culture

multidisciplinary collaboration theory

health systems strengthening

Integrating these frameworks would considerably strengthen the interpretation.

5. COREQ reporting

The manuscript appears generally consistent with qualitative reporting but several COREQ items remain unclear, including:

interviewer-participant relationship

repeat interviews

transcript verification

field notes

non-participation details

saturation process

A completed COREQ checklist should be carefully verified.

6. Sampling justification

Only 13 pharmacists participated.

Although this may be appropriate for qualitative work, the manuscript should better justify why this sample was sufficient beyond simply describing staff availability.

Minor Comments

Abstract

Avoid starting sentences with lowercase letters ("kidney transplant care...", "qualitative data...", "this study...").

Report the sampling strategy.

Mention thematic analysis according to Braun & Clarke.

Mention the number of hospitals in the Methods.

Introduction

The introduction is generally concise.

However,

include one paragraph explaining why pharmacists are particularly well positioned to evaluate transplant care delivery.

strengthen the novelty statement.

Methods

Several details require clarification.

State whether interviews were piloted.

Explain why verbal consent was preferred over written consent.

Describe translation procedures in greater detail.

State whether translations were independently checked.

Mention the software version of QDA Miner.

Results

The Results section is comprehensive.

However,

some quotations are excessively long.

Consider reducing the number of illustrative quotations while retaining representative examples.

Several quotations convey identical messages.

Figures

Figures 1 and 2 are visually appealing but largely conceptual summaries.

Figure 2 especially resembles a graphical summary rather than original analytical output.

Consider whether one conceptual figure would be sufficient.

Discussion

Several paragraphs could be shortened by 20–30%.

The discussion currently exceeds what is necessary for the findings presented.

Limitations

Please also acknowledge:

possible social desirability bias

limited transferability outside Baghdad

absence of physician and patient perspectives

possible translation bias

Language

Numerous grammatical inconsistencies remain throughout the manuscript.

Examples include:

inconsistent capitalization

"pharmacist role" vs "pharmacists' role"

"first of all"

"ultimately"

article usage

pluralization

verb tense consistency

Professional English editing is recommended.

**********

what does this mean?). If published, this will include your full peer review and any attached files.

If you choose “no”, your identity will remain anonymous but your review may still be made public.

**Do you want your identity to be public for this peer review?**  For information about this choice, including consent withdrawal, please see our Privacy Policy

Reviewer #1: No

**********

[NOTE: If reviewer comments were submitted as an attachment file, they will be attached to this email and accessible via the submission site. Please log into your account, locate the manuscript record, and check for the action link "View Attachments". If this link does not appear, there are no attachment files.]

To ensure your figures meet our technical requirements, please review our figure guidelines: https://journals.plos.org/plosone/s/figures

You may also use PLOS’s free figure tool, NAAS, to help you prepare publication quality figures: https://journals.plos.org/plosone/s/figures#loc-tools-for-figure-preparation.

NAAS will assess whether your figures meet our technical requirements by comparing each figure against our figure specifications.

# Author comment: Author response to Decision Letter 1

**DOI:** 10.1371/journal.pone.0360647.r002

4 Aug 2026

Responses to Academic Editor Comments:

Editor comments Author response

1. Improve methodological transparency and qualitative rigor Thank you for this valuable comment. We have revised the Methods section to improve methodological transparency and qualitative rigor. We expanded the description of participant recruitment, non-participation, interviewer reflexivity, interview setting, data saturation, transcription and translation procedures, coding and theme development, and ethical considerations. These revisions provide a more comprehensive description of the study procedures in accordance with the COREQ recommendations.

2. Strengthen the reflexivity statement In response to this comment. We expanded the reflexivity section by adding further information on the interviewer–participant relationship and the measures used to minimize interviewer and analytical bias. During the interviews, the first author periodically summarized and paraphrased participants’ responses to confirm understanding and seek clarification. In addition, the Arabic transcripts and their English translations were reviewed by the second author before coding, and coding decisions, theme development, and thematic refinement were discussed with the second author throughout the analysis until consensus on the final thematic structure was reached. The researcher’s professional background, which was already described in the original manuscript, was retained.

3. Strengthen the interpretation of findings Thank you for this valuable comment. The Discussion was revised to reduce repetition of the study findings and place greater emphasis on their interpretation. The possible reasons underlying the identified barriers and the relationships between them were further discussed, particularly in relation to organizational and health-system factors. Their potential implications for transplant care and multidisciplinary collaboration were also clarified where relevant.

4. Expand engagement with relevant qualitative literature Thank you for this important suggestion. The Discussion was revised to better integrate the existing literature with our findings rather than relying mainly on direct comparisons with previous studies. This allowed the findings to be discussed within a broader context, particularly in relation to organizational challenges, communication and collaboration, patient education, and medication-related system issues.

5. Review reporting against the COREQ checklist We appreciate the reviewer for highlighting the importance of comprehensive reporting according to the COREQ checklist. We carefully reviewed the manuscript against the COREQ guideline and revised the Methods section accordingly. All applicable COREQ items relevant to our study have now been addressed. As recommended, we clarified the audio-recording, transcription, translation, and verification process, as well as the procedures used to enhance the trustworthiness of the analysis. Items that were not undertaken (e.g., participant transcript verification/member checking, repeat interviews, and field notes) have not been reported to accurately reflect the study methods.

6. Better justify the sampling strategy We appreciate this valuable suggestion. We strengthened the justification for the sampling strategy by clarifying that all eligible pharmacists across the two participating transplant centers were invited to participate. The majority of eligible pharmacists participated, and information saturation was achieved, supporting the adequacy of the sample to address the study objectives.

7. Additional comments We thank the reviewer for these helpful additional comments. The manuscript has been revised accordingly.

* The Abstract has been revised accordingly.

* The Introduction has been strengthened by clarifying the rationale for selecting pharmacists as key informants and emphasizing the novelty of the study.

* The Methods section has been revised to address the reviewer’s comments.

* Participant quotations have been reduced where appropriate while maintaining adequate support for each theme.

* Figure 1 has been removed. Figure 2 has been retained because it provides an analytical synthesis of the findings.

* The Limitations section has been expanded to explicitly acknowledge potential social desirability bias and further clarify already listed ones.

* The Discussion was shortened, and language throughout the manuscript has been revised accordingly.

Responses to reviewer 1

Reviewer comments Author Responses

1.Insufficient reporting of qualitative rigor We appreciate the reviewer’s valuable comments. To address these concerns, we expanded the description of the qualitative analysis by clarifying how information saturation was determined, describing how differences in coding and interpretation were resolved through discussion until consensus was reached, and explaining that the coding framework and themes were iteratively reviewed and refined throughout the analysis until the final thematic structure was established.

2. Limited reflexivity In response to the reviewer’s valuable suggestion, we expanded the reflexivity section by clarifying the interviewer–participant relationship and describing how the interviewer’s professional background influenced participant rapport. We also added further details on the measures used to minimize potential interviewer and analytical bias during data collection and analysis, including the use of open-ended interviews, clarification of participants’ responses, and discussion of code and theme development with the second author throughout the analysis process.

3. Discussion is excessively descriptive We appreciate this important observation. We carefully revised and shortened the Discussion, particularly the sections that repeated findings already presented in the Results. We paid more attention to explaining the findings, the possible reasons behind the reported barriers, and how some of these challenges may lead to or worsen others. We also discussed their practical consequences for transplant care where relevant.

4. Greater engagement with qualitative literature Thank you for highlighting this point. We revised the Discussion to make better use of the available literature when interpreting our findings, rather than simply comparing whether similar findings had been previously reported. We used relevant evidence to provide further context for the challenges identified in this study and to support their interpretation within the healthcare setting.

5. COREQ reporting In response to this comment, we carefully reviewed the manuscript against the COREQ checklist and revised the Methods section accordingly. Additional details regarding the interviewer–participant relationship, non-participation, information saturation, and other relevant COREQ items have been incorporated where applicable.

6. Sampling justification In response to this comment, we further justified the sampling strategy by clarifying that all eligible pharmacists from the only two governmental kidney transplant centers in Baghdad were invited to participate. The majority of eligible pharmacists participated, and information saturation was achieved, supporting the adequacy of the sample for the study objectives.

7. Minor comments Thank you for these helpful comments. The manuscript was revised accordingly. The requested information was added to the Abstract, Introduction, and Methods. The Results and Discussion were shortened to reduce repetition, and Figure 1 was removed while Figure 2 was retained. The Limitations section was expanded as suggested, and the manuscript was carefully revised for language and consistency.

Attachment Submitted filename: response to reviewers.docx

# Peer review: Decision Letter 1

**DOI:** 10.1371/journal.pone.0360647.r003

**Authors:** Ali Haider Mohammed

31 Aug 2026

Dear Dr. Samer,

Thank you for submitting your manuscript to PLOS One. After careful consideration, we feel that it has merit but does not fully meet PLOS One’s publication criteria as it currently stands. Therefore, we invite you to submit a revised version of the manuscript that addresses the points raised during the review process.

Please submit your revised manuscript by  Oct 30 2026 11:59PM. If you will need more time than this to complete your revisions, please reply to this message or contact the journal office at plosone@plos.org. When you're ready to submit your revision, log on to https://www.editorialmanager.com/pone/ and select the 'Submissions Needing Revision' folder to locate your manuscript file.

- A letter that responds to each point raised by the academic editor and reviewer(s). You should upload this letter as a separate file labeled 'Response to Reviewers'.
- A marked-up copy of your manuscript that highlights changes made to the original version. You should upload this as a separate file labeled 'Revised Manuscript with Track Changes'.
- An unmarked version of your revised paper without tracked changes. You should upload this as a separate file labeled 'Manuscript'.

If you would like to make changes to your financial disclosure, please include your updated statement in your cover letter. Guidelines for resubmitting your figure files are available below the reviewer comments at the end of this letter.

If applicable, we recommend that you deposit your laboratory protocols in protocols.io to enhance the reproducibility of your results. Protocols.io assigns your protocol its own identifier (DOI) so that it can be cited independently in the future. For instructions see: https://journals.plos.org/plosone/s/submission-guidelines#loc-laboratory-protocols. Additionally, PLOS One offers an option for publishing peer-reviewed Lab Protocol articles, which describe protocols hosted on protocols.io. Read more information on sharing protocols at https://plos.org/protocols?utm_medium=editorial-email&utm_source=authorletters&utm_campaign=protocols.

As the corresponding author, your ORCID iD is verified in the submission system and will appear in the published article. PLOS supports the use of ORCID, and we encourage all coauthors to register for an ORCID iD and use it as well. Please encourage your coauthors to verify their ORCID iD within the submission system before final acceptance, as unverified ORCID iDs will not appear in the published article. *Only*  the individual author can complete the verification step; PLOS staff *cannot*  verify ORCID iDs on behalf of authors.

We look forward to receiving your revised manuscript.

Kind regards,

Ali Haider Mohammed

Academic Editor

PLOS One

Journal Requirements:

If the reviewer comments include a recommendation to cite specific previously published works, please review and evaluate these publications to determine whether they are relevant and should be cited. There is no requirement to cite these works unless the editor has indicated otherwise.

[Note: HTML markup is below. Please do not edit.]

Reviewers' comments:

Reviewer's Responses to Questions

**Comments to the Author**

Reviewer #1: All comments have been addressed

**********

2. Is the manuscript technically sound, and do the data support the conclusions?

Reviewer #1: Partly

**********

3. Has the statistical analysis been performed appropriately and rigorously?

Reviewer #1: Yes

**********

4. Have the authors made all data underlying the findings in their manuscript fully available?

The PLOS Data policy

Reviewer #1: No

**********

5. Is the manuscript presented in an intelligible fashion and written in standard English?

Reviewer #1: Yes

**********

Reviewer #1: The study is described generally as a “qualitative study,” and the authors state that Braun and Clarke’s thematic analysis was used. However, the manuscript does not identify the methodological orientation of the study, such as qualitative description, interpretive description, or another qualitative approach. It also does not explain whether the analysis was primarily inductive or deductive, semantic or latent, or whether the authors followed a reflexive, codebook, or coding-reliability form of thematic analysis.

The current description indicates that the first author completed the initial coding, after which codes and themes were reviewed with the second author and disagreements were resolved by consensus. This procedure may be appropriate, but the authors should name and justify the exact analytical approach rather than referring only broadly to Braun and Clarke.

The revised Methods should clearly explain:

The overall qualitative methodology and epistemological or theoretical orientation.

Whether coding was inductive, deductive, or a combination of both.

Whether the analysis focused on explicit meanings or underlying interpretations.

How initial codes were grouped, reviewed, and transformed into themes.

Whether a coding framework or codebook was developed and modified.

How differences between the two analysts were handled in practice.

How the authors determined that the final themes represented patterns of shared meaning rather than broad topic categories.

PLOS ONE requires qualitative analytical methods to be explicit, systematic, and sufficiently detailed, and recommends reporting according to COREQ or SRQR.

The interview guide covered pharmacists’ responsibilities, patient education, interprofessional collaboration, medication availability, barriers, and recommendations. The four final themes were pharmacist role, communication and collaboration, patient education, and medication availability.

This close overlap raises the possibility that the reported themes are largely summaries of the interview-guide domains rather than themes generated through deeper analysis of the data. This does not necessarily invalidate the findings, particularly in a qualitative descriptive study, but the authors must be transparent about the extent to which the analysis was guided by pre-existing interview domains.

The authors should:

Clarify whether the interview-guide domains formed an initial deductive coding structure.

Provide a coding tree or table showing the progression from quotations to codes, subthemes, and final themes.

Explain which findings emerged unexpectedly from the interviews.

Describe any contradictory, negative, or deviant cases.

Show more clearly how experiences differed between inpatient and outpatient pharmacists, between the two hospitals, and between less and more experienced pharmacists.

Consider whether the current four “descriptive themes” would be more accurately described as categories with analytical subthemes.

The manuscript would benefit from a stronger overarching interpretation, for example, whether informal adaptation by pharmacists compensates for but also potentially normalises structural fragmentation in transplant care.

The manuscript reports that 16 pharmacists were eligible, 13 participated, one declined, and two were unavailable because of extended leave. All 13 available pharmacists were interviewed, and the authors subsequently state that no substantially new codes or themes appeared in the final interviews.

This is better described as total-population or census sampling of all available eligible pharmacists, rather than a sample size determined by saturation. Saturation may have been observed, but it was not the reason recruitment stopped because all available eligible participants had already been interviewed.

Please clarify:

Whether saturation was assessed prospectively during data collection or retrospectively after all interviews.

After which interview no new codes were identified.

Who assessed saturation and how it was documented.

Whether the authors mean code saturation, meaning saturation, or general informational sufficiency.

Whether saturation was assessed separately for inpatient and outpatient pharmacists.

Whether the three non-participants differed meaningfully from participants in practice setting or experience.

The sample also included pharmacists with only one month of transplant experience, while the maximum experience was six years. The authors should justify why one month was considered sufficient exposure and discuss whether the accounts of recently appointed pharmacists differed from those of experienced pharmacists.

The study setting is central to the findings because the authors repeatedly attribute differences in communication and collaboration to whether a hospital had a dedicated transplant centre, the physical proximity of professionals, and the organisation of workflow. However, the Methods provide little information about the participating services beyond describing them as two public hospitals in Baghdad.

Without revealing hospital identities, the authors should report relevant contextual characteristics, such as:

Whether each hospital had a dedicated transplant ward or centre.

Approximate annual transplant or follow-up patient volume.

Number and type of pharmacists serving the transplant service.

Number of inpatient beds and outpatient dispensing workload.

Whether transplant surgery was performed at both hospitals.

How physician, nursing, and pharmacy services were structurally organised.

Whether medication procurement and dispensing systems differed between the hospitals.

The assertion that these are the “only two governmental hospitals” providing the relevant services in Baghdad should also be supported by an appropriate official or institutional source.

The authors state that Arabic transcripts were translated into English using an “AI-assisted translation tool,” reviewed against the original Arabic by the first author, and subsequently reviewed by the second author before coding. This is currently insufficient from both methodological and research-ethics perspectives.

The authors must report:

The exact name, provider, model or version, and access date of the AI translation tool.

Whether the tool was cloud-based, locally hosted, or institutionally licensed.

Whether transcripts were fully de-identified before being processed.

Whether the tool retained user data or used submitted content for model training.

Whether ethics approval and participant consent covered the use of an external AI service.

Whether potentially identifiable references to professionals, hospitals, or clinical incidents were removed before translation.

The language proficiency and translation qualifications of both authors.

How translation errors, culturally specific expressions, and ambiguous terminology were identified and resolved.

Whether coding was conducted from the Arabic transcripts, the English translations, or both.

Whether selected quotations were translated separately for publication.

PLOS requires authors to disclose the names of AI tools, how the tools were used, how their outputs were validated, and which parts of the study or data were affected.

This is an acceptance-critical issue. If identifiable participant material was uploaded to a third-party AI platform without appropriate consent, institutional approval, and confidentiality safeguards, the matter may require editorial or institutional ethics review.

The submission currently states only that “All data are in the manuscript file.” However, the manuscript contains selected quotations rather than the complete underlying qualitative dataset or an analytical dataset sufficient to evaluate the development of all reported themes.

PLOS requires the minimal data necessary to support reported findings to be available. When human-participant data cannot be shared publicly for ethical or confidentiality reasons, the Data Availability Statement must explain the restriction and identify how qualified researchers may request access.

Given the small and potentially identifiable professional population, unrestricted publication of full transcripts may not be ethical. Nevertheless, the authors should provide as much of the following as permitted:

The complete Arabic and English interview guides.

A de-identified coding framework or codebook.

Definitions and boundaries for each theme and subtheme.

A quotation-to-theme matrix using participant identifiers.

An analytical memo or table showing how themes were developed.

Additional de-identified supporting quotations.

A controlled-access procedure for fuller data, managed by a non-author institutional body such as the university ethics committee.

Where public transcript sharing is not possible, the statement should explain the re-identification risk, the consent limitations, the institutional body controlling access, and the conditions under which data requests would be reviewed.

The manuscript includes reports of patients missing doses after manufacturer changes and a participant’s statement that differences between anti-thymocyte globulin products had “in some cases” resulted in dosing errors. The Discussion also refers to duplicated dispensing and “medication leakage,” although the Results provide limited supporting evidence for these claims.

These are serious claims. The authors should clarify:

Whether these were documented incidents, direct observations, participant perceptions, or hypothetical risks.

How many participants reported each issue.

Whether dosing errors led to patient harm.

Whether these events were reported through formal pharmacovigilance or incident-reporting systems.

Whether “medication leakage” refers to duplicate dispensing, diversion, wastage, or another process.

Whether sufficient supporting quotations can be included without compromising confidentiality.

Throughout the Results and Discussion, wording should consistently indicate that these are pharmacists’ reported experiences or perceptions, unless independently verified. For example, “participants reported concerns that…” is preferable to wording that presents the event as an established system-level fact.

The manuscript now states that the first author was a pharmacist and master’s candidate, had no prior professional or supervisory relationship with participants, and that professional similarity facilitated rapport. This is useful, but reflexivity should extend beyond stating that potential bias was “minimized.”

Please discuss:

The second author’s professional background and relationship to the research topic.

How the authors’ assumptions about Iraqi pharmacy practice may have affected questioning, coding, and interpretation.

Whether the interviewer’s status as a pharmacist encouraged openness or generated socially desirable professional accounts.

Whether reflexive notes, analytical memos, or an audit trail were maintained.

How interpretations that challenged the researchers’ initial assumptions were handled.

Whether field notes, repeat interviews, transcript return, or participant checking were undertaken.

These procedures are not all mandatory. COREQ is intended to ensure transparent reporting rather than to prescribe that every technique must be performed. Activities that were not undertaken should therefore be stated transparently rather than simply omitted.

The study was limited to two hospitals in Baghdad and included pharmacists only. The authors themselves recognise that this limits transferability and prevents triangulation with physicians and patients. Nevertheless, the title refers broadly to “Iraqi pharmacists,” and the conclusion states that the findings indicate what is needed to strengthen “Iraqi transplant practices.”

The authors should avoid implying that the results represent Iraq nationally. A more accurate title would be:

Barriers and enablers shaping kidney transplant care delivery: Perspectives of pharmacists in two governmental transplant centres in Baghdad, Iraq

The abstract and conclusion should similarly refer to “the two participating Baghdad transplant centres” or “comparable governmental transplant settings,” rather than the entire Iraqi transplant system.

Although the Discussion has been shortened, it continues to repeat the Results and makes several interpretations that are more certain than the data permit. For example, the authors suggest that stable staffing “eventually led to improved medication availability,” that administrative work could “easily” be completed by non-pharmacists, and that pharmacists’ clinical development is comparable to that of physicians and nurses. These claims were not directly evaluated.

The Discussion should:

Distinguish clearly between study findings, authors’ interpretations, and proposed policy responses.

Avoid causal expressions such as “led to” when the data consist of cross-sectional participant accounts.

Remove repeated descriptions already presented in the Results.

Give greater attention to interactions among the themes.

Discuss why informal adaptations may be helpful in the short term but potentially fragile or unsafe as substitutes for formal systems.

Present recommendations as suggestions requiring implementation testing, rather than established solutions.

Avoid extrapolating from pharmacist perspectives to patient outcomes without patient-level data.

PLOS ONE requires interpretations and conclusions to be appropriately supported by the data and cautions against overstated conclusions.

**********

what does this mean?). If published, this will include your full peer review and any attached files.

If you choose “no”, your identity will remain anonymous but your review may still be made public.

**Do you want your identity to be public for this peer review?**  For information about this choice, including consent withdrawal, please see our Privacy Policy

Reviewer #1: No

**********

[NOTE: If reviewer comments were submitted as an attachment file, they will be attached to this email and accessible via the submission site. Please log into your account, locate the manuscript record, and check for the action link "View Attachments". If this link does not appear, there are no attachment files.]

To ensure your figures meet our technical requirements, please review our figure guidelines: https://journals.plos.org/plosone/s/figures

You may also use PLOS’s free figure tool, NAAS, to help you prepare publication quality figures: https://journals.plos.org/plosone/s/figures#loc-tools-for-figure-preparation.

NAAS will assess whether your figures meet our technical requirements by comparing each figure against our figure specifications.

# Author comment: Author response to Decision Letter 2

**DOI:** 10.1371/journal.pone.0360647.r004

23 Sep 2026

Reviewer comments Author responses

1. The revised Methods should clearly explain:

*The overall qualitative methodology and epistemological or theoretical orientation.

*Whether coding was inductive, deductive, or a combination of both.

*Whether the analysis focused on explicit meanings or underlying interpretations.

*How initial codes were grouped, reviewed, and transformed into themes.

*Whether a coding framework or codebook was developed and modified.

*How differences between the two analysts were handled in practice.

*How the authors determined that the final themes represented patterns of shared meaning rather than broad topic categories.

*PLOS ONE requires qualitative analytical methods to be explicit, systematic, and sufficiently detailed, and recommends reporting according to COREQ or SRQR.

Thank you for this important comment. We have revised the Methods section to provide a clearer description of the study methodology and analytical approach.

The study is now identified as using a qualitative descriptive approach, selected to provide a direct description of pharmacists’ perspectives and experiences. We have also clarified that Braun and Clarke’s reflexive thematic analysis was used.

The analysis was primarily inductive, with codes generated from the collected data rather than from a predefined coding framework, and was conducted at a semantic level, focusing on the explicit meanings reported by participants rather than latent meanings.

Although the broad areas of analysis were guided by the interview guide, the final theme names and content, as well as the subthemes, were developed from the collected data.

We also expanded the Methods section to describe the analytical process more clearly. We also clarified the role of the second author.

*COREQ is provided as a supplementary file

2. The interview guide covered pharmacists’ responsibilities, patient education, interprofessional collaboration, medication availability, barriers, and recommendations. The four final themes were pharmacist role, communication and collaboration, patient education, and medication availability.

Clarify whether the interview-guide domains formed an initial deductive coding structure.

We appreciate this valuable notice. We clarified that although the broad areas of analysis were guided by the interview guide, they did not form a predefined deductive coding structure. Initial codes were generated inductively from the collected data, while the final theme names, content and subthemes were developed through the analysis.

Provide a coding tree or table showing the progression from quotations to codes, subthemes, and final themes.

Thank you for this suggestion. We have added a supplementary analytical table showing the progression from representative quotations to example codes, subthemes, and final themes. As no formal coding tree was developed during the analysis, the table is presented as an illustrative analytical structure rather than a retrospective codebook.

Explain which findings emerged unexpectedly from the interviews We agree that distinguishing findings that arose from the analysis from the areas anticipated in the interview guide would improve transparency.

We clarified this point at the beginning of the Results section, noting that while the themes remained related to the areas the study intended to explore, the analysis also generated additional findings not predefined by the interview guide.

Describe any contradictory, negative, or deviant cases. Thank you for this important comment. We reviewed the Results to ensure that contrasting, less commonly reported, and differing perspectives were adequately represented rather than limiting the findings to the dominant views. Across themes, we retained and reported variation in participants’ experiences and practices where present.

Within the pharmacist-role theme, we presented the dominant dispensing- and administrative-focused role alongside the experiences of pharmacists who described undertaking additional activities beyond their formally assigned responsibilities.

In the collaboration theme, we explicitly described differences between inpatient and outpatient pharmacists and between the two hospitals. The findings also included both facilitating and challenging aspects of communication, as well as perspectives reported by only a small number of participants. To further strengthen the representation of such differing perspectives, we added the concern expressed by one pharmacist regarding overreliance on informal communication methods, despite these methods being described by other participants as useful adaptive strategies.

We also retained variation within the patient-education theme. Although most pharmacists described providing patient education, the content and method of education were not consistent across participants. Some pharmacists provided broader education beyond medication-related information, while educational delivery varied between verbal counseling, written instructions, and indirect education through nursing staff. Similarly, not all inpatient pharmacists reported discharge counseling, further illustrating variation in practice rather than a uniform approach.

For medication availability and access, the findings did not reveal directly opposing views; instead, participants described different problems according to their individual experiences and practice settings. For example, outpatient experiences included patient concerns or reluctance when medication manufacturers changed, whereas inpatient experiences highlighted potential confusion among healthcare staff arising from differences between available products. We therefore retained these different experiences rather than presenting an artificial contradiction when the data did not support it.

Show more clearly how experiences differed between inpatient and outpatient pharmacists, between the two hospitals, and between less and more experienced pharmacists. Thank you for this important comment. We reviewed the Results to ensure that differences between inpatient and outpatient pharmacists and between the two hospitals were clearly represented, and these were already reflected across the relevant themes.

However, differences related to pharmacists’ level of experience were less explicit. Therefore, we revised the pharmacist-role theme to highlight that several evolving practices were described by more experienced pharmacists. This pattern is also further interpreted in the Discussion in relation to accumulated experience and stable staffing.

Consider whether the current four “descriptive themes” would be more accurately described as categories with analytical subthemes. Thank you for this helpful suggestion. We considered whether the four overarching findings would be more appropriately described as categories. However, after reviewing the thematic structure, we retained the term “themes,” as each represents a recurring pattern across participants’ accounts rather than only a broad topic corresponding to the interview-guide domains.

Although the broad areas were anticipated in the interview guide, the final theme names and content reflect the patterns identified through the analysis. The revisions made throughout the Results were also intended to make these patterns and the variation within each theme more explicit.

The manuscript would benefit from a stronger overarching interpretation Thank you for this helpful suggestion. We agree that a stronger overarching interpretation would improve the integration of the study findings. Therefore, we added a concluding paragraph to the Discussion that brings the four themes together and highlights the broader pattern across them.

3. Please clarify:

*Whether saturation was assessed prospectively during data collection or retrospectively after all interviews.

*After which interview no new codes were identified.

*Who assessed saturation and how it was documented.

*Whether the authors mean code saturation, meaning saturation, or general informational sufficiency.

*Whether saturation was assessed separately for inpatient and outpatient pharmacists.

*Whether the three non-participants differed meaningfully from participants in practice setting or experience. Thank you for this important clarification. We agree that recruitment in this study is more accurately described as total-population sampling rather than as being determined by saturation. Therefore, recruitment ended because all available eligible pharmacists had been interviewed, rather than because a predetermined saturation threshold had been reached.

We have revised the Methods accordingly and removed the previous statement regarding saturation.

Regarding the three non-participants, two were outpatient pharmacists, and one was an inpatient pharmacist. The two outpatient pharmacists were not senior staff, and pharmacists with comparable practice settings and experience were represented among the participants.

Although the non-participating inpatient pharmacist held a more senior role, three other inpatient pharmacists from the same hospital participated in the study, including pharmacists with substantial experience.

The sample also included pharmacists with only one month of transplant experience, while the maximum experience was six years. The authors should justify why one month was considered sufficient exposure and discuss whether the accounts of recently appointed pharmacists differed from those of experienced pharmacists. Thank you for raising this point. No minimum duration of transplant experience was specified because the study aimed to reflect pharmacists’ perspectives and experiences as they existed in routine practice.

Pharmacists with limited experience were assigned to the transplant service and had direct contact with transplant patients. In fact, they were the only inpatient pharmacists available in that hospital during the study period. Therefore, excluding them solely based on duration of experience would have excluded an important aspect of the actual practice setting.

Their inclusion also provided insight into the experiences of recently appointed pharmacists, including challenges related to limited practical experience.

4. *The study setting is central to the findings because the authors repeatedly attribute differences in communication and collaboration to whether a hospital had a dedicated transplant centre, the physical proximity of professionals, and the organisation of workflow. However, the Methods provide little information about the participating services beyond describing them as two public hospitals in Baghdad.

*The assertion that these are the “only two governmental hospitals” providing the relevant services in Baghdad should also be supported by an appropriate official or institutional source. Thank you for this comment. We expanded the Study Setting section to provide further contextual information about the two hospitals.

We also clarified the important differences in outpatient service organization between the hospitals, particularly regarding dedicated transplant facilities, physician availability, pharmacy organization, and the physical proximity of services. These contextual details provide additional background for interpreting the differences between the two settings identified in the findings.

Regarding the statement that these were the only two governmental hospitals providing kidney transplant services in Baghdad, we were unable to identify an official published source that could verify this information, as it was based on verbal information available to the researchers. Therefore, to avoid making an unsupported claim, we revised the wording from “the only two” to “two major.”

5. *The authors state that Arabic transcripts were translated into English using an “AI-assisted translation tool,” reviewed against the original Arabic by the first author, and subsequently reviewed by the second author before coding. This is currently insufficient from both methodological and research-ethics perspectives.

*PLOS requires authors to disclose the names of AI tools, how the tools were used, how their outputs were validated, and which parts of the study or data were affected. *Thank you for this important comment. We have revised the Methods to provide further details regarding the AI-assisted translation process.

The interviews were manually transcribed verbatim and de-identified by the first author before translation. ChatGPT (OpenAI) was used only as an assistive translation tool, and successive de-identified transcript excerpts were entered rather than complete transcript files. No participant names, hospital identities, or other identifying information were submitted to the tool.

*The first author, an MSc candidate in Pharmacy with good English proficiency (IELTS overall score 7.5), reviewed each translated section against the original Arabic transcript and corrected translation errors, context-specific expressions, and terminology where necessary. The Arabic and English transcripts were subsequently reviewed by the second author before coding.

*Coding was conducted using the English-translated transcripts. The quotations reported in the manuscript were taken directly from these translations and were not translated separately for publication.

*ChatGPT is a cloud-based service. The specific model variant used at the time was not recorded; however, the translations were conducted using the GPT-5.2 family available at that time

*The ethics approval and consent did not specifically address the use of an external AI service; however, AI-assisted translation was performed only after complete de-identification, and no identifiable participant or institutional information was submitted.

6. PLOS requires the minimal data necessary to support reported findings to be available. When human-participant data cannot be shared publicly for ethical or confidentiality reasons, the Data Availability Statement must explain the restriction and identify how qualified researchers may request access.

Thank you for this comment. We have revised the Data Availability Statement to clarify that all data that can be shared without compromising confidentiality are included in the manuscript and Supporting Information. The complete interview transcripts cannot be shared because of the risk of re-identification and because participant consent did not include permission for sharing interview data with other researchers.

7. The manuscript includes reports of patients missing doses after manufacturer changes and a participant’s statement that differences between anti-thymocyte globulin products had “in some cases” resulted in dosing errors. The Discussion also refers to duplicated dispensing and “medication leakage,” although the Results provide limited supporting evidence for these claims.

The authors should clarify:

*Whether these were documented incidents, direct observations, participant perceptions, or hypothetical risks.

*How many participants reported each issue.

*Whether dosing errors led to patient harm.

*Whether these events were reported through formal pharmacovigilance or incident-reporting systems.

*Whether “medication leakage” refers to duplicate dispensing, diversion, wastage, or another process.

*Whether sufficient supporting quotations can be included without compromising confidentiality.

Throughout the Results and Discussion, wording should consistently indicate that these are pharmacists’ reported experiences or perceptions Thank you for this important comment. We reviewed the relevant findings and revised both the Results and Discussion to more clearly distinguish between commonly reported experiences and isolated participant observations.

The quotations relating to ATG-related concentration differences and possible dosing errors, as well as the quotation describing possible missed doses following manufacturer changes, were already included in the manuscript.

We have now clarified the frequency of these findings in the Results, including that patient concern and confusion were reported by six pharmacists, while missed doses and dosing errors were each reported by individual participants.

We also added a supporting quotation for concerns regarding possible duplicate dispensing and clarified that this issue was raised by two pharmacists. The terminology was revised to avoid implying that duplicate dispensing was a formally documented event, and the previous term “medication leakage” was removed.

Finally, the Results and Discussion were revised for consistency, with more cautious wording throughout to indicate that these findings reflect pharmacists’ reported observations and perceptions. We could not determine from the available data whether the reported dosing or dispensing incidents resulted in patient harm or were formally documented; therefore, no such claims are made in the revised manuscript.

8. The manuscript now states that the first author was a pharmacist and master’s candidate, had no prior professional or supervisory relationship with participants, and that professional similarity facilitated rapport. This is useful, but reflexivity should extend beyond stating that potential bias was “minimized.”

Please discuss:

*The second author’s professional background and relationship to the research topic.

*How the authors’ assumptions about Iraqi pharmacy practice may have affected questioning, coding, and interpretation.

*Whether the interviewer’s status as a pharmacist encouraged openness or generated socially desirable professional accounts.

*Whether reflexive notes, analytical memos, or an audit trail were maintained.

*How interpretations that challenged the researchers’ initial assumptions were handled.

*Whether field notes, repeat interviews, transcript return, or participant checking were undertaken.

Thank you for this comment. Several of the points raised had already been addressed in the previous revision. The manuscript already reports that the first author, who conducted the interviews, was a pharmacist and MSc candidate with no prior professional or supervisory relationship with participants.

It also describes the use of summarizing and paraphrasing during interviews to confirm understanding, the iterative review of interpretations during analysis, and acknowledges the potential influence of the first author’s professional background in the Limitations.

In the current revision, we have additionally clarified the role of the second author. He is an academic clinical pharmacist with a PhD in clinical Pharmacy and published more than 3 qualitative studies. He served as the research supervisor who supervised and revised every step. He was not involved in recruitment or interviewing, had no direct contact with participants during data collection, and was not introduced to them as part of the research team at that stage.

We also confirm that no field notes, repeat interviews, transcript return, formal participant checking, reflexive memos, or formal audit trail were undertaken. These procedures are therefore reported as not performed rather than being retrospectively inferred.

9. *The study was limited to two hospitals in Baghdad and included pharmacists only. The authors themselves recognise that this limits transferability and prevents triangulation with physicians and patients. Nevertheless, the title refers broadly to “Iraqi pharmacists,” and the conclusion states that the findings indicate what is needed to strengthen “Iraqi transplant practices.”

The authors should avoid implying that the results represent Iraq nationally.

*The abstract and conclusion should similarly refer to “the two participating Baghdad transplant centres” or “comparable governmental transplant settings,” rather than the entire Iraqi transplant system.

Thank you for this important observation. We agree that the findings should not be interpreted as nationally representative, and we have therefore revised the wording in the Abstract and Conclusion to more clearly reflect the two participating governmental hospitals in Baghdad and to avoid extrapolating the findings to transplant practice across Iraq.

However, we respectfully retained the current title. The phrase “Iraqi pharmacists” is intended to describe the nationality and professional background of the participating pharmacists rather than to imply national representativeness. The geographical and institutional scope of the study is clearly specified in the Methods and Limitations, where the study is explicitly described as being conducted in two governmental hospitals in Baghdad.

10. Although the Discussion has been shortened, it continues to repeat the Results and makes several interpretations that are more certain than the data permit

The Discussion should:

*Distinguish clearly between study findings, authors’ interpretations, and proposed policy responses.

*Avoid causal expressions such as “led to” when the data consist of cross-sectional participant accounts.

*Remove repeated descriptions already presented in the Results.

*Give greater attention to interactions among the themes.

*Discuss why informal adaptations may be helpful in the short term but potentially fragile or unsafe as substitutes for formal systems.

*Present recommendations as suggestions requiring implementation testing, rather than established solutions.

*Avoid extrapolating from pharmacist perspectives to patient outcomes without patient-level data. Thank you for this important comment. We revised both the Results and Discussion to more clearly separate descriptive findings from interpretation.

Interpretive wording was reduced in the Results, while repeated descriptions were removed from the Discussion and replaced with more focused interpretation and linkage across themes.

We also removed or softened causal and overly certain statements that were not directly supported by the data, including claims regarding staffing, administrative duties, and comparisons with physicians and nurses.

Informal adaptations are now discussed as useful but potentially fragile substitutes for formal systems, and recommendations are presented as potential strategies whose feasibility and effectiveness require evaluation before wider implementation.

We also avoided extending pharmacists’ perspectives to patient outcomes where patient-level data were not available.

Attachment Submitted filename: Response_to_reviewers_auresp_2.docx

# Editor report: Decision Letter 2

**DOI:** 10.1371/journal.pone.0360647.r005

**Authors:** Ali Haider Mohammed

29 Sep 2026

Barriers and Enablers Shaping Kidney Transplant Care Delivery: Insights from Iraqi Pharmacists

PONE-D-26-26371R2

Dear Dr. Samer,

We’re pleased to inform you that your manuscript has been judged scientifically suitable for publication and will be formally accepted for publication once it meets all outstanding technical requirements.

Within one week, you’ll receive an e-mail detailing the required amendments. When these have been addressed, you’ll receive a formal acceptance letter and your manuscript will be scheduled for publication.

An invoice will be generated when your article is formally accepted. Please note, if your institution has a publishing partnership with PLOS and your article meets the relevant criteria, all or part of your publication costs will be covered. Please make sure your user information is up-to-date by logging into Editorial Manager at Editorial Manager® and clicking the ‘Update My Information' link at the top of the page. For questions related to billing, please contact billing support.

If your institution or institutions have a press office, please notify them about your upcoming paper to help maximize its impact. If they’ll be preparing press materials, please inform our press team as soon as possible -- no later than 48 hours after receiving the formal acceptance. Your manuscript will remain under strict press embargo until 2 pm Eastern Time on the date of publication. For more information, please contact onepress@plos.org.

Kind regards,

Ali Haider Mohammed

Academic Editor

PLOS One

Additional Editor Comments (optional):

Reviewers' comments:

# Editor report: Acceptance letter

**DOI:** 10.1371/journal.pone.0360647.r006

**Authors:** Ali Haider Mohammed

PONE-D-26-26371R2

PLOS One

Dear Dr. Mohammed,

I'm pleased to inform you that your manuscript has been deemed suitable for publication in PLOS One. Congratulations! Your manuscript is now being handed over to our production team.

At this stage, our production department will prepare your paper for publication. This includes ensuring the following:

* All references, tables, and figures are properly cited

* All relevant supporting information is included in the manuscript submission,

* There are no issues that prevent the paper from being properly typeset

You will receive further instructions from the production team, including instructions on how to review your proof when it is ready. Please keep in mind that we are working through a large volume of accepted articles, so please give us a few days to review your paper and let you know the next and final steps.

Lastly, if your institution or institutions have a press office, please let them know about your upcoming paper now to help maximize its impact. If they'll be preparing press materials, please inform our press team within the next 48 hours. Your manuscript will remain under strict press embargo until 2 pm Eastern Time on the date of publication. For more information, please contact onepress@plos.org.

You will receive an invoice from PLOS for your publication fee after your manuscript has reached the completed accept phase. If you receive an email requesting payment before acceptance or for any other service, this may be a phishing scheme. Learn how to identify phishing emails and protect your accounts at https://explore.plos.org/phishing.

If we can help with anything else, please email us at customercare@plos.org.

Thank you for submitting your work to PLOS One and supporting open access.

Kind regards,

PLOS One Editorial Office Staff

on behalf of

Dr. Ali Haider Mohammed

Academic Editor

PLOS One
